# Physics-Guided Multi-View Transformer with Block Attention Residuals for Cross-Condition Railway Bearing Fault Diagnosis

**DOI:** 10.3390/s26206439

**Published:** 2026-10-12

**Authors:** Junjie Liang, Guoqiang Cai, Zhengming Zhang, Shuqin Lin, Yuxiao Song, Zihao Wu

**Affiliations:** State Key Laboratory of Advanced Rail Autonomous Operation, Beijing Jiaotong University, Beijing 100044, China; 24120760@bjtu.edu.cn (J.L.); 24120902@bjtu.edu.cn (Z.Z.); 24120763@bjtu.edu.cn (S.L.); 24120814@bjtu.edu.cn (Y.S.); 24120845@bjtu.edu.cn (Z.W.)

**Keywords:** railway bearing, fault diagnosis, cross-condition, physics-guided multi-view transformer

## Abstract

Railway bearings experience speed- and load-dependent distribution shifts that complicate fault diagnosis. This study proposes a Physics-Guided Multi-View Transformer with Block Attention Residuals (PGMV-BAR). Characteristic-order prior tokens sequentially query envelope-order and time–frequency features and remain in the fusion sequence with a separate condition token. A two-stage strategy combines masked reconstruction, contrastive consistency, prior alignment, and supervised fine-tuning. Evaluation follows a source-only cross-condition protocol. Beijing Jiaotong University–Rail Autonomous Operations (BJTU-RAO) includes nine combinations of motor frequency (20, 40, and 60 Hz) and lateral load (−10, 0, and +10 kN), with a constant vertical load of 10 kN. All 36 unordered pairs are evaluated as held-out test conditions, with the remaining seven conditions supplying source training and validation in each task. The L dataset is evaluated through six leave-one-condition-out tasks formed by 1200, 1500, and 1800 r/min at 0% and 10% rated torque, using the other five conditions as sources. Held-out-condition samples are excluded from pretraining, fine-tuning, and checkpoint selection. PGMV-BAR achieves a mean accuracy of 97.27% across the 36 BJTU-RAO tasks (96.05–99.21%) and 95.33% across the L tasks. These results support cross-condition diagnosis within the evaluated datasets.

## 1. Introduction

Reliable bearing fault diagnosis is essential for railway equipment operating under varying speeds and loads. Changes in operating conditions affect the frequencies, amplitudes, and temporal distributions of vibration components, complicating the transfer of diagnostic models between conditions. The Beijing Jiaotong University–Rail Autonomous Operations (BJTU-RAO) bogie datasets provide a relevant experimental setting for investigating these challenges [1]. A central issue is how to preserve fault-related information while accounting for operating-condition-dependent variations in measured signals.

Deep learning has expanded the available approaches to this problem. Liu et al. combined convolutional and bidirectional recurrent representations with attention for bearing diagnosis in train bogie transmission systems [2]. Zhu et al. investigated multilevel time–frequency integration and multiscale feature fusion [3]. More recently, Wang et al. developed a dual-branch interactive fusion network using weighted differential time–frequency features, combining convolutional feature extraction with Swin Transformer modeling [4]. Yan et al. proposed MGFR-ViT, which integrates local enhancement and multiscale gated refinement into a Vision Transformer [5]. These studies demonstrate that complementary feature interaction and the joint modeling of local and global information are established design strategies. The distinction sought in the present work therefore concerns how bearing-kinematic priors participate in these interactions, rather than the use of multiple branches or Transformer components alone.

Cross-condition learning introduces an additional requirement: a representation must remain informative when operating conditions change. Li et al. explored Transformer-based meta-learning for bearing identification under multiple small-sample conditions [6]. Song et al. combined covariance loss and graph convolutional networks for domain generalization [7]. Zhao and Yang proposed working-condition-indexed generative domain generalization, using operating parameters to guide data generation for unseen conditions [8]. Their work establishes the relevance of explicitly representing operating conditions, although it relies on a generation pathway guided by the specified condition index. In the present study, operating information instead enters the diagnostic fusion sequence as a condition token. These approaches address related distribution shifts through different computational roles for condition information.

Recent machinery health-monitoring research also extends beyond conventional fault classification. Zhou et al. proposed a knowledge library network for non-exemplar incremental remaining useful life prediction [9]. Wang et al. investigated cross-type bearing diagnosis using a contrastive cluster zero-shot model [10], while Qi et al. explored mechanical degradation assessment using TimeGPT [11]. These studies provide relevant perspectives on knowledge reuse, transfer, and temporal representation learning. Their prediction targets and supervision settings differ, however, from the cross-condition classification task considered here. They consequently inform the methodological context without serving as directly interchangeable performance baselines.

Physically meaningful signal processing and hybrid learning provide further foundations. Zhou et al. transformed acoustic-emission signals into continuous-wavelet-transform images and used a deep residual network for source localization [12]. This illustrates how signal representations informed by the measurement process can support learning, without necessarily imposing governing equations on the network. Selami et al. combined statistical descriptors extracted from raw signals and empirical mode decomposition components with an IGWO-optimized neural classifier [13]. This approach emphasizes compact feature engineering and classifier optimization, making the selected descriptors central to the information available for diagnosis. The present work instead retains spectral and time–frequency feature sequences for subsequent interaction with explicit prior representations.

Physical knowledge has also been incorporated directly into attention and cross-condition learning. Cheng et al. developed AFARN, which uses bearing fault characteristics to guide attention and aligns source and target feature distributions [14]. Jian et al. developed a physics-informed semi-supervised domain generalization framework that combines semantic feature learning with displacement prediction constrained by governing dynamic equations [15]. Physical consistency and prediction uncertainty guide pseudo-label weighting and contrastive alignment using labeled and unlabeled source-domain samples, with target-domain data excluded during training. Its physical constraint therefore relies on the adopted dynamics formulation, while its semi-supervised strategy exploits available unlabeled source observations. PGMV-BAR investigates a different role for physical information: nominal characteristic-order tokens sequentially collect spectral and time–frequency evidence and remain available for joint fusion. Shi and Wang further combined characteristic-frequency normalization with physics-guided spectral attention for cross-speed bearing diagnosis [16].

The sensing arrangement also determines the information available to a diagnostic model. Sim et al. developed a physics-guided dual-sensor framework that uses a spatially separated reference sensor and a selected carrier band to identify consistent transmitted vibration components [17]. Its physical guidance depends on the relationship between measurement locations. The two views used in the present work are instead derived from the same vibration segment and represent complementary spectral and time–frequency information. This distinction separates additional sensing information from complementary representations of an existing measurement.

The preceding review identifies a specific architectural question concerning the role of physical knowledge in multi-view learning. Physically informed preprocessing can organize the input, and prior-guided attention can influence feature weighting. A further possibility is to represent nominal fault periodicities as explicit queries that accumulate evidence across observation views. The resulting design question is how characteristic-order queries can first identify relevant spectral information, use that information to guide a subsequent interaction with time–frequency features, and retain the accumulated evidence for joint diagnosis. This question concerns the evolution and subsequent use of the prior representation, providing a concrete basis for distinguishing the proposed framework from feature fusion alone.

To address this question, this study proposes a Physics-Guided Multi-View Transformer with Block Attention Residuals (PGMV-BAR). Its central architectural contribution is a sequential interaction mechanism built around characteristic-order prior tokens. These tokens first query the encoded envelope order spectrum, producing prior representations conditioned on the measured spectral evidence. The updated representations then query the encoded time–frequency observations, allowing the second interaction to depend explicitly on the information collected in the first. The final prior tokens are retained alongside both observation streams and a separate operating-condition token for joint fusion. Spectral prior-alignment regularization provides a physical reference for the first interaction, while Block Attention Residuals, adopted from the Attention Residuals framework [18], support aggregation across fusion blocks. This architecture gives the physical priors a continuing role in evidence collection and fusion.

The contributions of this work are summarized as follows:

- A sequential prior-query mechanism is developed for multi-view bearing diagnosis. Characteristic-order tokens first acquire spectral evidence and then use their updated representations to query time–frequency features, establishing an explicit dependence between the two stages of physical-prior interaction.
- An architecture is formulated that retains the observation-updated prior tokens alongside both observation streams for joint fusion. Spectral prior-alignment regularization and a separate condition token provide complementary physical and operating context for this architecture, linking nominal fault periodicities, measured evidence, and operating descriptors through distinct computational roles.
- The framework is evaluated on the BJTU-RAO and self-collected L datasets through cross-condition experiments, baseline comparisons, and controlled component analyses. These evaluations examine diagnostic performance, the contributions of the constituent pathways, and the applicability of the physical guidance under the investigated conditions.

The remainder of this paper is organized as follows. Section 2 presents the theoretical foundations. Section 3 describes PGMV-BAR, its optimization objectives, implementation details, and evaluation metrics. Section 4 presents the baseline models, experimental platforms, datasets, and preprocessing. Section 5 reports the results and discusses their interpretation and limitations. Section 6 concludes the paper.

## 2. Theoretical Foundations

### 2.1. Bearing Fault Characteristic Orders

Local defects in rolling bearings follow explicit kinematic frequency laws. The theoretical characteristic frequencies of four typical local faults follow the standard bearing-kinematic relationships [19] and are expressed as

$$f_{o}=\frac{Z}{2}f_{r}(1-\frac{d_{b}}{D_{p}}\cos\alpha )$$

$$f_{i}=\frac{Z}{2}f_{r}(1+\frac{d_{b}}{D_{p}}\cos\alpha )$$

$$f_{c}=\frac{1}{2}f_{r}(1-\frac{d_{b}}{D_{p}}\cos\alpha )$$

(1) $$f_{b}=\frac{D_{p}}{2d_{b}}f_{r}\left[1-(\frac{d_{b}}{D_{p}}\cos\alpha {)}^{2}\right]$$

where *f*_o_, *f*_i_, *f*_c_, and *f*_b_ denote the outer-race, inner-race, cage, and rolling-element characteristic frequencies, respectively. *Z* is the number of rolling elements. *d*_b_, *D*_p_, *α*, and *f*_r_ are the rolling-element diameter, pitch diameter, contact angle, and rotational frequency, respectively.

The fault characteristic frequency is jointly determined by the structural parameters and rotational frequency. Variations in operating conditions shift spectral peaks and further increase intra-class dispersion. To mitigate this effect, the order representation is introduced as

(2) $$O=\frac{f}{f_{r}}$$

where *O* is the order and *f* is the frequency.

Under ideal conditions, samples from the same fault type remain concentrated around similar order neighborhoods under different operating conditions. Therefore, order representation is naturally suitable for cross-condition feature alignment and semantic preservation.

The actual rotational frequencies of bearings at different locations are not identical. Gearbox bearings are usually directly associated with the drive-side rotational speed, whereas axle-box bearings are affected by the transmission chain. Therefore, the bearing location and gear ratio should be considered when calculating characteristic orders.

Accordingly, the fault characteristic frequencies and orders determined by structural parameters, rotational-speed relationships, and location-dependent transmission characteristics constitute the theoretical basis for fault-mechanism analysis and are further used as physical priors in the proposed model.

### 2.2. Multi-View Feature Construction

#### 2.2.1. Spectral Representation

Local fault impacts usually excite high-frequency resonances, whereas the periodic fault rhythm is mainly embedded in the resonance envelope.

Let *x*_b_(*t*) be the signal after filtering in the fault-sensitive frequency band. Its envelope is obtained using Hilbert-envelope analysis [19] and is defined as

$$e(t)=\left|x_{b}(t)+jH\{x_{b}(t)\}\right|$$

where *e*(*t*) is the envelope signal, and H(.) denotes the Hilbert transform.

The frequency-domain representation of the envelope signal is then obtained as

E(f)=F(e(t))

where *E*(*f*) is the envelope spectrum, and F(.) denotes the Fourier transform.

The envelope order spectrum is further constructed as

(3) $$S_{O}(O)=\mathrm{Interp}(\left|E(f)\right|)$$

where *S*_O_(*O*) is the envelope order spectrum, and Interp(.) denotes frequency-to-order interpolation.

This process reparameterizes the spectral fault structure. The frequency drift driven by speed variation is converted into a relatively stable order-alignment problem, and a fixed-length envelope order spectrum is finally constructed as the spectral input.

#### 2.2.2. Time–Frequency Representation

Although order reparameterization improves the stability of the fault rhythm, it remains a globally compressed representation and cannot reveal the nonuniform excitation of local impacts over time. Since fault energy often appears as localized aggregation, drift, and intermittent enhancement in the time–frequency plane, a joint representation with both temporal and frequency resolutions is required.

The short-time Fourier transform (STFT) [20] is employed for time–frequency processing of vibration signals. The time–frequency transform is written as

$$X(t,f)=\int_{-\infty }^{\infty }x(\tau )w(\tau -t)e^{-j2\pi f\tau }d\tau$$

where *X*(*t*, *f*) is the STFT coefficient, *w*(.) is the window function, and *t* is time.

After magnitude compression and normalization, a time–frequency representation matrix is obtained to describe the local textures and evolution paths of fault energy in the time–frequency plane.

### 2.3. Transformer Encoder

The Transformer [21] uses attention as its core mechanism to aggregate contextual information by modeling global correlations among inputs. Given an input ***X***, the query, key, and value matrices are defined as

$$Q_{i}=XW_{i}^{Q},K_{i}=XW_{i}^{K},V_{i}=XW_{i}^{V}$$

$W_{i}^{Q}$, $W_{i}^{K}$, and $W_{i}^{V}$ are learnable projection matrices for query, key, and value, respectively; *i* is the attention head. The scaled dot-product attention is written as

$$\mathrm{Attention}(Q,K,V)=\mathrm{Softmax}(\frac{QK^{T}}{\sqrt{d_{k}}})V$$

where *d*_k_ is the key dimension.

To enhance feature representation, the Transformer further adopts the following multi-head attention formulation:

$$\mathrm{MultiHead}(X)=\mathrm{Concat}({\mathrm{head}}_{1},\ldots ,{\mathrm{head}}_{h})W_{o}$$

$${\mathrm{head}}_{i}=\mathrm{Attention}(Q_{i},K_{i},V_{i})$$

where ***W***_o_ is the output projection matrix, and *h* is the number of heads.

Because the Transformer does not contain recurrent or convolutional structures, positional encoding is required to preserve sequence-position information. Each attention module is typically followed by a position-wise feedforward network, with residual connections and layer normalization applied around both the attention and feedforward modules.

**Figure 1:**
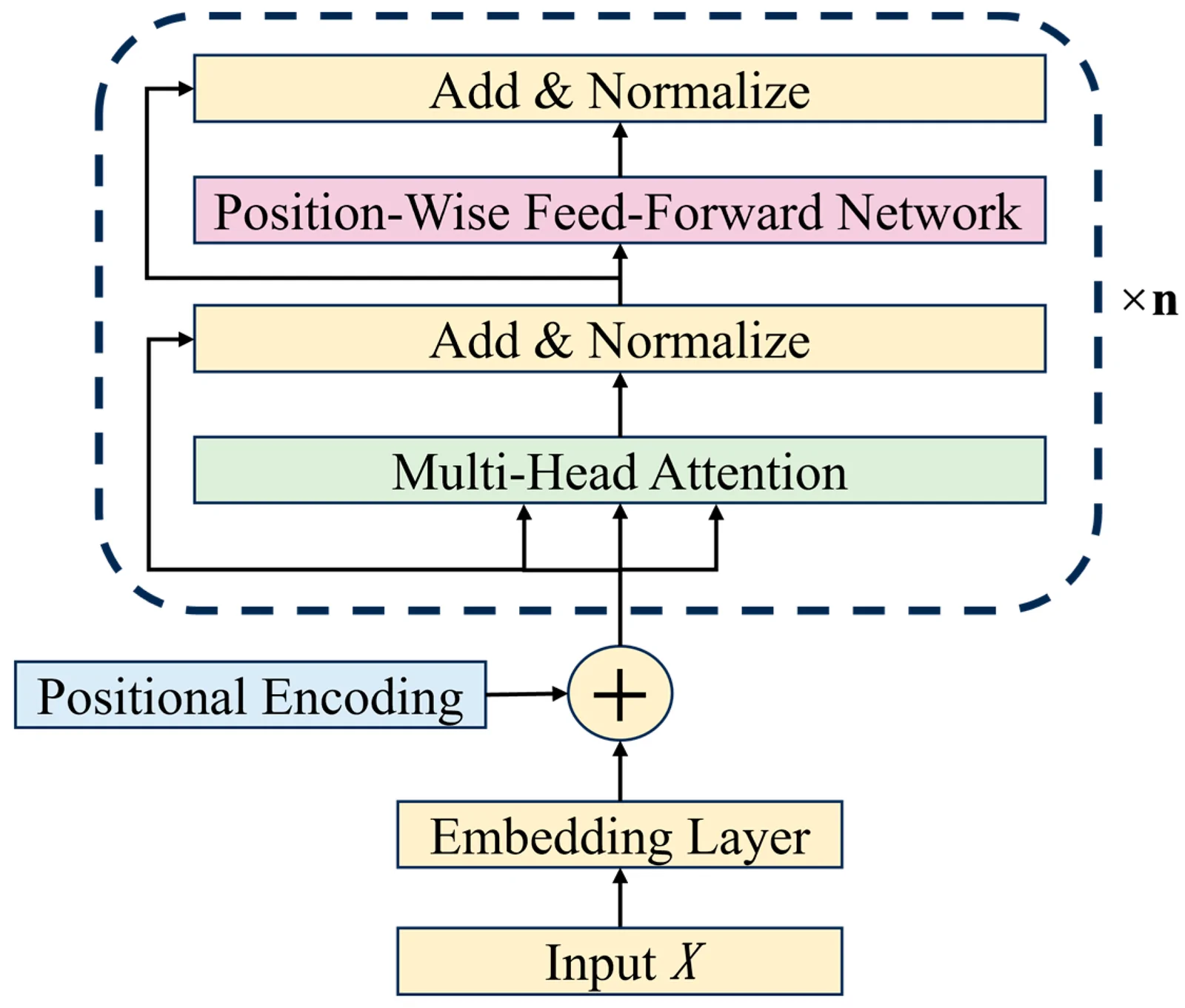
Schematic architecture of the Transformer encoder.

The task considered in this study is bearing fault classification. The final class prediction is obtained from the classification head applied to the encoder output. Figure 1 illustrates the encoder structure; the decoding and reconstruction branches support auxiliary training objectives and are omitted from this schematic.

### 2.4. Attention Residuals

To stabilize the training of deep networks, the Transformer adopts residual learning. However, a standard residual connection essentially performs fixed and equally weighted accumulation of previous-layer outputs, and cannot adaptively adjust the contributions of different depths according to the input content. As the network becomes deeper, early-layer information may be progressively diluted, while the current layer cannot explicitly access specific historical-layer representations.

To overcome the fixed accumulation of standard residuals along the depth dimension, Kimi Team [18] proposed Attention Residuals, which replace residual addition with an attention-based depth-propagation mechanism.

Let the input of layer *l* be a weighted sum of historical depth representations. Then

(4) $$H_{l}=\sum_{i=0}^{l-1}\alpha_{i\to l}V_{i}$$

where ***H****_l_* is the layer *l* input, ***V****_i_* is the value at depth position *i*, and *α_i_*_→*l*_ is the depth-attention weight.

Let ***q****_l_* be the depth-query vector of layer *l* and ***k****_i_* be the key representation of the historical position *i*. The similarity score is defined as

(5) $$s_{i\to l}=\varphi (q_{l},k_{i})$$

The corresponding attention weight is

(6) $$\alpha_{i\to l}=\frac{\exp(\varphi (q_{l},k_{i}))}{\sum_{j=0}^{l-1}\exp(\varphi (q_{l},k_{j}))}$$

and satisfies $\sum_{i=0}^{l-1}\alpha_{i\to l}=1$. In Attention Residuals, the layer *l* usually corresponds to a learnable pseudo-query vector ***w****_l_*, i.e., ***q****_l_* = ***w****_l_*.

Thus, information propagation along the depth dimension is no longer determined by fixed residual addition. Instead, it is driven by learnable depth attention, enabling the current layer to adaptively rely on shallow, intermediate, or deep representations according to the input content.

Applying attention aggregation to all preceding layers incurs high storage and communication costs. To address this issue, [18] further proposed Block Attention Residuals (BAR). The main idea is to divide *L* layers into *N* blocks, preserve standard residual accumulation within each block, and perform attention aggregation only across blocks. Let $B_{n}$ be the layer-index set of block *n*. Its block-level representation is written as

(7) $$b_{n}=\sum_{j\in B_{n}}G_{j}(H_{j})$$

where ***b****_n_* is the block *n* representation, $B_{n}$ is its layer-index set, ***H****_j_* is the hidden state, and *G_j_*(.) is the layer *j* transformation.

If the partial sum of the first *i* sublayers within a block is further defined, then

(8) $$b_{n}^{i}=\sum_{j\in B_{n}^{i}}G_{j}(H_{j})$$

where $b_{n}^{i}$ is the partial sum in block *n*, and $B_{n}^{i}$ is the corresponding index subset.

Consequently, the depth-value set accessible to the current layer is no longer composed of all historical single-layer outputs, but consists of historical block representations and the partial sum of the current block. Let this set be $V_{n}$. The current-layer input can be uniformly represented as

(9) $$H_{l}=\sum_{r=1}^{n}\alpha_{r\to l}V_{r},V_{r}\in V_{n}$$

where ***V****_r_* is a block or partial-block representation, and *α_r→l_* is the block-level attention weight.

**Figure 2:**
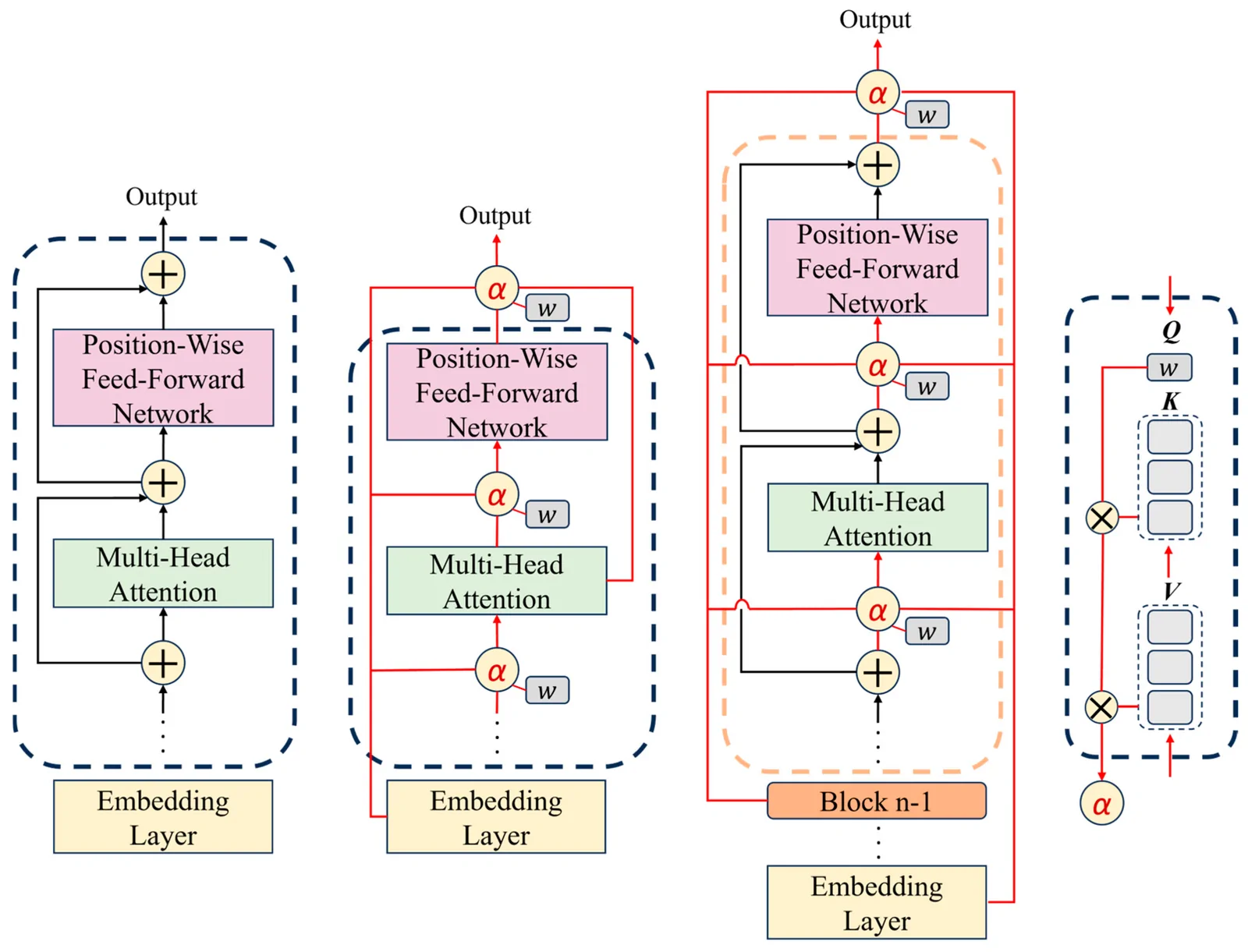
Residual connection mechanisms and attention-based weighting: standard residual connections; full attention residual connections; block attention residual connections; attention-based residual weighting mechanism. Green and purple boxes denote multi-head attention and position-wise feedforward networks, respectively. Pale-yellow boxes denote embedding layers, and the orange box represents a preceding block. Black lines indicate standard forward and residual connections, whereas red lines indicate depth-attention connections. Dark-blue dashed outlines group computational components, and the orange dashed outline identifies the current block. Circled plus and multiplication signs denote addition and multiplication, respectively. Circles labeled α indicate depth-attention weighting, and w denotes a learnable pseudo-query. BAR converts the depth-wise information flow from fixed linear accumulation to selective weighted aggregation, thereby retaining cross-layer information-selection capability while controlling the additional overhead.

The three residual connection mechanisms are illustrated in Figure 2.

## 3. Proposed Method

The PGMV-BAR framework consists of four components: dual-view input embedding, prior-guided interaction encoding, fusion encoding, and classification output. The core idea is to highlight fault-sensitive information using dual-view inputs constrained by physical mechanisms, anchor fault semantics through interactions between prior tokens and multi-view features, and enhance cross-layer information propagation using a BAR fusion encoder, thereby improving diagnostic stability under complex operating conditions.

After preprocessing, an input vibration sample is represented by the envelope order spectrum ***X***_s_ and the time–frequency representation ***X***_tf_. The corresponding physical prior vector is ***p***, and the operating-condition vector is ***c***. The overall mapping of the proposed model is summarized as

(10) $$\hat{y}=f(X_{s},X_{\mathrm{tf}},p,c;Q)$$

where $\hat{y}$ is the predicted class-probability vector, and Q is the parameter set.

### 3.1. Dual-View Input and Prior Encoding

#### 3.1.1. Spectral View

The spectral branch takes the envelope order spectrum as input. After discretization, the spectral input vector is $X_{s}\in {\mathbb{R}}^{L_{s}}$, where *L*_s_ is the spectral sequence length.

To adapt the spectral sequence to the Transformer input format, it is divided into fixed-length patches and projected into the embedding space:

$$T_{s}={\mathrm{PatchEmbed}}_{s}(X_{s})+E_{s}$$

where $T_{s}\in {\mathbb{R}}^{N_{s}\times d}$ is the spectral-token sequence, *N*_s_ is the token number, *d* is the embedding dimension, and ***E***_s_ is the positional encoding.

#### 3.1.2. Time–Frequency View

The time–frequency branch takes the STFT-based time–frequency matrix as input. The matrix is $X_{\mathrm{tf}}\in {\mathbb{R}}^{H\times W}$, where *H* is the frequency dimension, and *W* is the time dimension of the time–frequency map.

Similarly, the time–frequency map is divided into two-dimensional patches and embedded into a unified feature space:

$$T_{\mathrm{tf}}={\mathrm{PatchEmbed}}_{\mathrm{tf}}(X_{\mathrm{tf}})+E_{\mathrm{tf}}$$

where $T_{\mathrm{tf}}\in {\mathbb{R}}^{N_{\mathrm{tf}}\times d}$ is the time–frequency-token sequence, *N*_tf_ is the token number, and ***E***_tf_ is the two-dimensional positional encoding.

#### 3.1.3. Prior and Condition Encoding

To introduce bearing-kinematic prior information into the model, the theoretical characteristic orders are constructed as the physical prior vector ***p*** = [*O*_o_, *O*_i_, *O*_c_, *O*_b_], where *O*_o_ is the theoretical order of the outer-race fault, *O*_i_ is the theoretical order of the inner-race fault, *O*_c_ is the theoretical order of the cage fault, and *O*_b_ is the theoretical order of the rolling-element fault.

The prior vector is further mapped into prior tokens through a multilayer perceptron:

$$T_{p}={\mathrm{MLP}}_{p}(p)+E_{p}$$

where $T_{p}\in {\mathbb{R}}^{N_{p}\times d}$ is the prior-token representation, *N*_p_ is the token number, and ***E***_p_ is the prior-token embedding.

Four prior tokens are used, one for each nominal fault order. Each scalar order is mapped by a 1–384–384 MLP with GELU activation and a learned prior-type embedding, producing a 4 × 384 prior representation per sample. Here, “physical” refers to the kinematic origin of the input orders, not to a hard constraint on the learned embedding. For fixed bearing geometry and a consistent shaft-speed reference, the nominal orders are speed-independent under the assumptions in Section 2.1.

Operating conditions are represented by dataset-specific dimensionless descriptors and mapped to a common condition-token architecture. For BJTU-RAO, the descriptors are the motor-speed ratio, vertical-load ratio, and lateral-load ratio, forming the vector $c_{\mathrm{BJTU}}=[r_{s},r_{v},r_{h}]$. The reference values are 40 Hz for motor rotational frequency and 10 kN for both vertical and lateral loads. The three ratios are defined in Equation (11).

(11) $$r_{s}=\frac{f_{m}}{f_{m}^{\mathrm{ref}}},r_{v}=\frac{L_{v}}{L_{v}^{\mathrm{ref}}},r_{h}=\frac{L_{h}}{L_{h}^{\mathrm{ref}}}$$

where *f*_m_ and $f_{m}^{\mathrm{ref}}$ are the current and reference motor rotational frequencies, respectively; *L*_v_ and *L*_h_ are the current vertical and lateral loads; $L_{v}^{\mathrm{ref}}$ and $L_{h}^{\mathrm{ref}}$ are the corresponding reference loads.

For the L dataset, the operating descriptors are shaft speed and torque loading. Let *n* denote rotational speed in r/min, *f*_r_ *= n/*60 the corresponding frequency in Hz, and *q = T/T*_rated_ the applied torque fraction, where *T*_rated_ = 5 N·m. The condition vector is constructed as $c_{L}=\left[\frac{f_{r}}{f_{\mathrm{ref}}},\frac{q}{q_{\mathrm{ref}}},\frac{q}{q_{\mathrm{ref}}}\right]$, *f*_ref_ = 40 Hz, *q*_ref_ = 0.1.

The two load entries encode the same dimensionless torque fraction, preserving the three-dimensional input interface. They do not represent vertical or lateral forces. The 0% and 10% torque settings therefore yield normalized load entries of 0 and 1, respectively. The reference values are fixed configuration constants. Speed and torque descriptors are supplied from operating metadata during both training and testing.

For both datasets, a 3–384–384 MLP with GELU activation maps the continuous descriptor vector to one 384-dimensional condition token. A bearing-location embedding and a learned condition-token-type embedding are added to the MLP output. In the L dataset, all samples share the same location identifier. No discrete operating-condition-ID embedding is used. The condition token supplies operating context during fusion, while the physical prior tokens represent nominal bearing fault periodicities.

#### 3.1.4. Prior-Guided Interaction

After the dual-view tokens are obtained, they are fed into the spectral and time–frequency encoders for branch-specific feature extraction:

$$H_{s}={\mathrm{Enc}}_{s}(T_{s}),H_{\mathrm{tf}}={\mathrm{Enc}}_{\mathrm{tf}}(T_{\mathrm{tf}})$$

where $H_{s}\in {\mathbb{R}}^{N_{s}\times d}$ and $H_{\mathrm{tf}}\in {\mathbb{R}}^{N_{\mathrm{tf}}\times d}$ are the spectral-branch and time–frequency-branch outputs, respectively.

A standard multi-head cross-attention module is used to condition the prior representation on observed evidence. The initial prior tokens first query the spectral features, and the resulting spectrum-conditioned prior tokens subsequently query the time–frequency features. The two updates are expressed as

(12) $$\begin{array}{l} {\tilde{T}}_{p\to s}={\mathrm{CA}}_{s}(T_{p},H_{s},H_{s})\\ {\tilde{T}}_{p\to \mathrm{tf}}={\mathrm{CA}}_{\mathrm{tf}}({\tilde{T}}_{p\to s},H_{\mathrm{tf}},H_{\mathrm{tf}}) \end{array}$$

Here, CA_s_ and CA_tf_ denote the complete prior-to-spectrum and prior-to-time–frequency interaction blocks, respectively. Their first, second, and third arguments supply the query, key, and value representations.

The interaction proceeds through two sequential updates of the prior representation. Each block uses eight attention heads with an embedding dimension of 384, layer-normalized queries and keys/values, a residual attention update, and a residual feedforward sublayer. The second interaction therefore depends on the spectral evidence acquired during the first. The final updated prior tokens are retained in the fusion sequence in Equation (13).

This sequential update gives the prior tokens a continuing computational role across the two views. Although the nominal characteristic orders can be shared by samples with the same bearing configuration, the updated tokens depend on the measured features of each sample. The tokens therefore provide a route through which spectral evidence influences the subsequent retrieval of time–frequency information. Retaining these tokens during fusion makes the accumulated prior-conditioned evidence available together with the two observation streams. The spectral prior-alignment objective in Section 3.3.2 supplies an additional physical reference for the first interaction. The resulting mechanism combines physically anchored queries with observation-dependent updates; the attention operations themselves remain standard, and the learned embeddings are not subject to a hard kinematic constraint.

### 3.2. Fusion Encoder

After dual-branch encoding and the two sequential prior-guided interactions, the classification token, condition token, spectral features, time–frequency features, and final updated prior tokens are concatenated to form the fusion input sequence:

(13) $$Z_{0}=[t_{\mathrm{cls}};t_{c};H_{s};H_{\mathrm{tf}};{\tilde{T}}_{p\to \mathrm{tf}}]$$

Here, the first two entries denote the classification and condition tokens, respectively; the last entry is the prior-token sequence after both interactions in Equation (12); and the semicolons denote concatenation along the token dimension.

BAR is introduced into the fusion encoder. Let *L*_f_ be the total number of fusion-encoder layers, and *S*_b_ be the block size. The number of blocks is *N*_b_ = *L*_f_/*S*_b_.

According to the block-partition strategy reported in [18] and the model configuration used in this study, the fusion encoder contains 16 layers organized into eight blocks of two layers, with embedding dimension 384 and feedforward expansion ratio 4.0. The spectral and time–frequency branches each contain six standard encoder layers without BAR. At block boundaries, the block representations are retained for attention-based aggregation; the current partial block and stored blocks provide the depth values. Learned pseudo-queries and normalized keys determine the aggregation weights, following the adopted BAR formulation [18].

For the BJTU-RAO spectral input, 768 order points and 32-point patches produce 24 tokens. For L, 384 order points produce 12 tokens with the same patch length. Time–frequency features use 8 × 8 patches; their token count is floor(*H*/8) × floor(*W*/8), with incomplete edge patches excluded. The fusion sequence contains one classification token, one condition token, the two observation-token sequences, and four updated prior tokens. The processing sequence is window construction → two-view feature extraction → branch encoding → spectral prior query → time–frequency prior query → joint BAR fusion → classification.

### 3.3. Output and Optimization

#### 3.3.1. Classification Output

After the fusion encoder, the global representation corresponding to the classification token, ***h***_cls_, is fed into the classification head for fault identification:

$$\hat{y}=\mathrm{Softmax}(W_{c}h_{\mathrm{cls}}+b_{c})$$

where ***W***_c_ and ***b***_c_ are the classifier weight and bias, respectively.

#### 3.3.2. Pretraining Objective

To improve representation learning under limited training samples, auxiliary pretraining objectives are introduced at the early training stage, including masked reconstruction loss, contrastive consistency loss, and prior alignment loss. The total pretraining objective is formulated as

(14) $$L_{\mathrm{pre}}=\lambda_{1}L_{\mathrm{mask}}+\lambda_{2}L_{\mathrm{con}}+\lambda_{3}L_{\mathrm{prior}}$$

where $L_{\mathrm{mask}}$, $L_{\mathrm{con}}$, and $L_{\mathrm{prior}}$ are the masked reconstruction, contrastive consistency, and prior alignment losses, respectively, and $\lambda_{1}-\lambda_{3}$ are their weights.

The masked reconstruction target is the unmasked, preprocessed patch vector from each input view. Let $M_{v}$ be the selected masked-patch indices of view *v*, *D_v_* the number of values in each patch, and *x* and $\hat{x}$ the target and reconstructed values. The masking ratio is 0.1, and the losses of the spectral and time–frequency views are summed:

$$L_{\mathrm{mask}}=\sum_{v\in \{\mathrm{spec},\mathrm{tf}\}}\frac{1}{|M_{v}|D_{v}}\sum_{j\in M_{v}}\sum_{d=1}^{D_{v}}\rho \left({\hat{x}}_{vjd}-x_{vjd}\right)$$

Here, $\rho (u)=\left\{\begin{array}{ll} \frac{1}{2}u^{2}, & |u|<1,\\ |u|-\frac{1}{2}, & |u|\geq 1. \end{array}\right.$ A view with no masked patches contributes zero.

The contrastive objective uses two independently augmented versions of each source-training window. Their normalized 64-dimensional projections form a positive pair. Projections from the other windows in the minibatch are negatives; fault labels are not used to construct the pairs. For a batch of *B* windows, *p(i)* denotes the paired augmentation of anchor *i*. The normalized temperature-scaled contrastive loss [22] is

$$L_{\mathrm{con}}=-\frac{1}{2B}\sum_{i=1}^{2B}\log\frac{\exp\left(z_{i}^{T}z_{p(i)}/\tau \right)}{\sum_{\begin{array}{c} k=1\\ k\neq i \end{array}}^{2B}\exp\left(z_{i}^{T}z_{k}/\tau \right)},\;\;\tau =0.2$$

The prior-alignment objective acts on the prior-to-spectrum attention. Let *a_bpj_* denote the attention for sample *b*, prior *p*, and spectral token *j* after averaging the eight heads and normalizing over spectral tokens. A Gaussian target *g_bpj_* is centered on the nominal fundamental order *o_bp_* at the token-center order *c_j_*:

$$g_{bpj}=\frac{\exp\left[-\frac{{\left(c_{j}-o_{bp}\right)}^{2}}{2\sigma^{2}}\right]}{\sum_{k=1}^{N_{s}}\exp\left[-\frac{{\left(c_{k}-o_{bp}\right)}^{2}}{2\sigma^{2}}\right]},\;\;\sigma =0.35$$

The Gaussian width is expressed in order units.

$$L_{\mathrm{prior}}=\frac{1}{B}\sum_{b=1}^{B}\sum_{p=1}^{4}\sum_{j=1}^{N_{s}}g_{bpj}\log\left(\frac{g_{bpj}}{a_{bpj}}\right)$$

The sum over the four priors is retained, and averaging is over the batch. Attention values are clamped to 10^−6^ before normalization and logarithms. This is KL(target ‖ attention), not a label-conditioned loss, and the target does not explicitly include harmonic peaks.

Pretraining was performed for 30 epochs using only the retained source-training subset. The masked reconstruction, contrastive, and prior-alignment weights were 0.1, 0.05, and 0.1, respectively. Supervised fine-tuning used the same retained source-training subset. Source-validation samples and held-out-condition samples were excluded from both optimization stages. During pretraining, the classification-loss term was disabled; labels were used for stratified subset construction and diagnostic reporting, but not for classification supervision. All four nominal priors were determined by the bearing configuration rather than selected from the true fault label.

Input standardization and resonance-band selection were performed separately for each window. For BJTU-RAO, channel ranking was performed separately within each original recording. For L, the supplied acceleration TXT records were processed directly as one-dimensional signals, without channel ranking. At testing, the same rules were applied to the held-out input; no pooled source–target normalization transform was fitted, and target-derived features or statistics were not fed back into training. Source validation was used only for hyperparameter and checkpoint selection.

#### 3.3.3. Fine-Tuning Objective

During fault classification, the supervised classification objective is combined with the prior-alignment term. A class-weighted focal loss [23] with label smoothing is used to account for differences in sample difficulty and class distribution. For sample *i*, let *y_i_* denote the true class and *p_i,c_* the predicted probability of class *c*. The smoothed target is defined as

$$q_{i,c}=(1-\varepsilon )1(c=y_{i})+\frac{\varepsilon }{c},\;\;\varepsilon =0.05,$$

where *C* is the number of classes and 1(⋅) denotes the indicator function. The classification loss is

(15) $$L_{\mathrm{cls}}=-\frac{1}{B}\sum_{i=1}^{B}\alpha_{y_{i}}[\max(1-p_{i,y_{i}},10^{-6}){]}^{\gamma }\sum_{c=1}^{C}q_{i,c}\log p_{i,c}$$

where *B* is the minibatch size, $\alpha_{y_{i}}$ is the weight of the true class calculated from the retained source-training labels, and *γ* = 2. Label smoothing applies to the cross-entropy target, while the focal modulation and class weight remain determined by the true class. The lower bound of 10^−6^ follows the numerical implementation of the focal factor. This classification objective is used during supervised fine-tuning.

The final fine-tuning objective is

(16) $$L_{\mathrm{fine}}=L_{\mathrm{cls}}+\lambda_{3}L_{\mathrm{prior}}$$

Fine-tuning optimized the supervised classification objective together with the prior-alignment term using the same retained source-training windows as pretraining. Class weights were computed from the retained training labels. Training augmentation was enabled only for training inputs, whereas validation and test inputs used deterministic preprocessing. An exponential moving average (EMA) with decay 0.999 was used for source validation and checkpoint selection.

**Figure 3:**
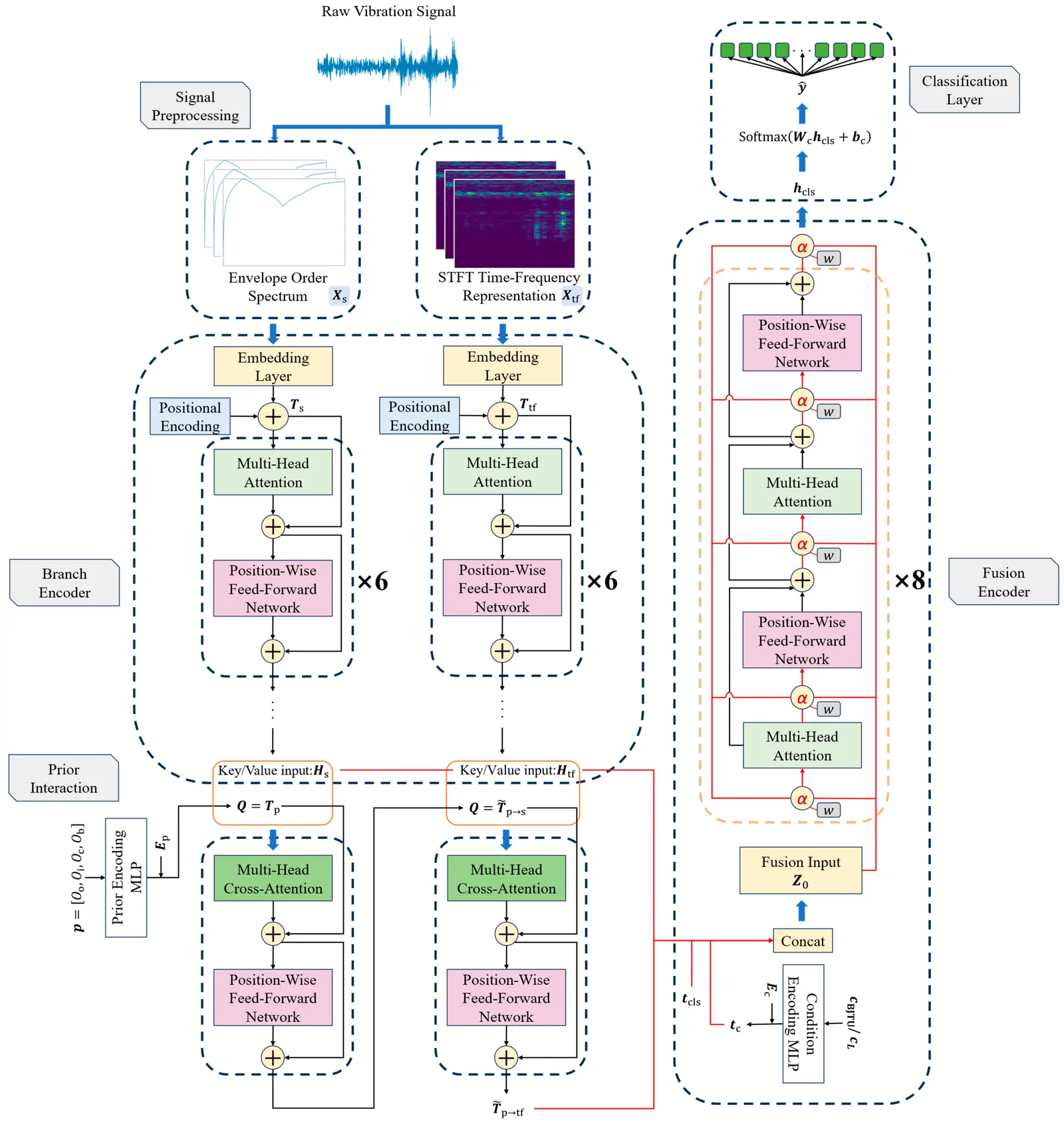
Framework of the proposed PGMV-BAR. Shared graphical conventions follow those described in Figure 2. Light-blue boxes denote positional encoding, and blue arrows indicate the main processing flow. Pale-yellow boxes additionally represent concatenation and fusion-input operations. Red lines also indicate connections supplying features and tokens for concatenation. The labels ×6 and ×8 denote six encoder layers in each observation branch and eight two-layer blocks in the fusion encoder, respectively.

The overall diagnostic workflow of the proposed PGMV-BAR is shown in Figure 3.

### 3.4. Implementation Details and Evaluation Metrics

#### 3.4.1. Implementation Details

Multi-dataset experiments were conducted on BJTU-RAO and the self-collected L dataset to assess cross-condition bearing fault diagnosis. BJTU-RAO provided the principal evaluation across speed–load combinations, and L provided a separate evaluation with another bearing and acquisition system. Each evaluated model–task configuration was independently trained and evaluated using random seeds 53, 54, and 55. Results are reported as mean ± sample standard deviation across the three runs. For each run, checkpoint selection used source-validation performance, and the selected checkpoint was subsequently evaluated on the held-out conditions.

For PGMV-BAR, hyperparameter selection followed a staged adjustment strategy based on source-validation performance. Candidate configurations were compared with respect to diagnostic performance, convergence stability, and training cost.

PGMV-BAR training comprised 30 auxiliary pretraining epochs followed by at most 100 supervised fine-tuning epochs. AdamW [24] used a batch size of 16, an initial learning rate of 2 × 10^−5^, and weight decay of 5 × 10^−4^. Fine-tuning used 10 warmup epochs followed by cosine annealing to a minimum learning-rate ratio of 0.1. The embedding dimension, attention heads, branch depth, fusion depth, and dropout were 384, 8, 6, 16, and 0.15, respectively. Table 1 gives the remaining settings. All data-dependent model selection used source validation.

The model was implemented in PyTorch version 2.11.0+cu130. All experiments were performed on a workstation with an NVIDIA RTX PRO 6000 Blackwell Workstation Edition GPU, an Intel Xeon Platinum 8488C CPU, 125 GB RAM, and a Linux operating system.

Computational profiling was performed for the eight-block BAR, four-block BAR, and no-BAR configurations using the BJTU-RAO input settings in Section 4.2. Measurements used the workstation described above, FP32, and a batch size of one. Estimated FLOPs counted the principal operations covered by the profiler using two FLOPs per MAC. Network latency was measured over 500 repetitions per configuration using the complete forward pass, including the auxiliary heads. The reported network latency therefore characterizes this complete-forward configuration. Processing-pipeline latency included CPU preprocessing, data transfer, model execution, and prediction return, excluding signal acquisition and disk reading, and was measured over 150 repetitions. Latency standard deviations describe timing variability rather than variation across training seeds. Randomly initialized models were used for structural profiling.

#### 3.4.2. Evaluation Metrics

Overall accuracy, macro-precision, macro-recall, macro-F1, and class-wise recall were used as the main evaluation metrics. Overall accuracy is calculated as Accuracy = *N*_correct_/*N*, where *N*_correct_ is the number of correct predictions, and *N* is the number of test samples.

For fault type *x*, precision, recall, and F1-score are calculated as follows:

$$P_{x}=\frac{{\mathrm{TP}}_{x}}{{\mathrm{TP}}_{x}+{\mathrm{FP}}_{x}}$$

$$R_{x}=\frac{{\mathrm{TP}}_{x}}{{\mathrm{TP}}_{x}+{\mathrm{FN}}_{x}}$$

$$F1_{x}=\frac{2P_{x}R_{x}}{P_{x}+R_{x}}$$

where TP*_x_*, FP*_x_*, and FN*_x_* are the true-positive, false-positive, and false-negative counts of class *x*, respectively.

For multiclass classification, macro-precision, macro-recall, and macro-F1 are calculated as follows:

$$\mathrm{Macro-Precision}=\frac{1}{C}\sum_{x=1}^{C}P_{x}$$

$$\mathrm{Macro-Recall}=\frac{1}{C}\sum_{x=1}^{C}R_{x}$$

$$\mathrm{Macro-F}1=\frac{1}{C}\sum_{x=1}^{C}F1_{x}$$

where *C* is the number of classes.

For the repeated classification experiments, metrics were calculated separately for seeds 53, 54, and 55 and summarized as mean ± sample standard deviation, using *n* − 1 in the variance denominator. The BJTU prior-sensitivity analysis and the L-dataset error and occlusion analyses used fixed trained checkpoints and are reported separately from these across-seed summaries. Overall performance was obtained by averaging task results within each seed before aggregation across seeds. Baseline comparisons paired accuracy differences by held-out task and seed; these differences were averaged across tasks within each seed and summarized across the three seeds. Task-wise win/tie/loss counts used three-seed mean accuracies. Two-sided exact sign-flip tests exchanged model labels jointly across all tasks within each seed, with Holm adjustment across the six baseline comparisons separately for each dataset. This analysis assessed variation across seeds on the fixed evaluation tasks. Confusion matrices and t-SNE visualizations provided complementary analyses of predictions and learned representations.

## 4. Experimental Work

### 4.1. Baseline Models

To evaluate diagnostic performance, six representative baselines were implemented: 1D-CNN [25], MpResCNN-BiLSTM-Attention [2], TF-ResNet based on the SE-ResNet-50 branch in [26], TF-Swin-Transformer adapted from [27], Spec+TF Dual-Branch Fusion inspired by [3] and [28], and PFN–PGSA [16]. All six baselines were evaluated on both BJTU-RAO and the L dataset using their respective condition-holdout protocols.

The six baselines were implemented as explicitly specified adaptations to the present diagnostic protocol. The 1D-CNN uses four convolutional stages with channel widths of 32, 64, 128, and 192 and kernel sizes of 15, 9, 7, and 5. MpResCNN-BiLSTM-Attention uses three cascaded multiscale residual streams with kernel sizes of 7, 15, and 31, followed by a bidirectional LSTM with 96 hidden units per direction and temporal attention pooling. TF-ResNet uses a single-channel SE-ResNet-50 backbone with bottleneck stage depths of [3,4,6]. TF-Swin-Transformer uses a single-channel Swin-T configuration with stage depths of [2,6], attention heads of [3,6,12,24], an initial embedding dimension of 96, a patch size of 4, and a window size of 7. Spec+TF Dual-Branch Fusion combines a spectral MLP with hidden dimensions of 256 and 128 with a time–frequency CNN having channel widths of 32, 64, and 96. PFN–PGSA uses a 128-bin BPFI-normalized log-amplitude spectrum and a single 64-dimensional, four-head physics-guided attention block, with a feed-forward expansion ratio of four and mean pooling. Its physical mask incorporates bearing-specific inner-race, outer-race, and rolling-element frequency ratios with four harmonics, inverse-harmonic weights, and Gaussian kernels with a standard deviation of two bins. The unnormalized mask outer product is mixed with data-dependent attention through a learned sigmoid coefficient. The resulting features are combined with the common operating-condition representation for nine-class BJTU-RAO or five-class L classification. Target-dependent adaptation objectives are excluded, and this implementation is reported as a source-only adaptation of the physical-frequency-normalization and spectral-attention components in [16].

All compared methods follow the dataset-specific condition-holdout, window-generation, sample-retention, and waveform-normalization procedures described in Section 4.2 and Section 4.3. Time-domain baselines receive standardized waveforms, time–frequency baselines receive single-channel STFT representations, and the dual-branch baseline receives envelope order spectra together with STFT representations. These transformations use the corresponding PGMV-BAR preprocessing settings, and STFT inputs retain their native dimensions without RGB conversion or image resizing. PFN–PGSA uses the characteristic-frequency-normalized log-amplitude spectrum described above, preserving its method-specific input representation.

All six baselines receive the continuous operating descriptors defined in Section 3.1.3. A two-layer MLP with dimensions 3–384–384 and a GELU activation encodes the descriptors. The resulting representation is combined with a site embedding and a learned type bias, concatenated with the pooled signal features, and passed to a classifier with a 384-unit hidden layer, GELU activation, and dropout of 0.15. BJTU-RAO uses the gearbox/axle-box site information, whereas L uses a constant site identifier. The duplicated load entries for L encode the same torque fraction. Discrete condition identifiers and fault labels are not predictive inputs. The baselines do not incorporate the proposed characteristic-order query tokens or the PGMV-BAR prior-alignment objective. PFN–PGSA instead uses its own harmonic-mask-based attention regularization.

Training augmentation is applied to the waveform before representation construction. It comprises amplitude scaling by a factor sampled uniformly from 0.92 to 1.08; Gaussian noise with probability 0.65 and a standard deviation equal to 2% of the current signal standard deviation; time stretching within 0.99–1.01 with probability 0.20; and local signal masking with probability 0.10. The original window length is restored, followed by normalization. Augmentation is reproducible for each sample and random seed. Validation and test samples use deterministic preprocessing without augmentation.

The baselines use fixed optimization settings without a separate model-specific hyperparameter search. Supervised training lasts at most 100 epochs, with batch size 16, AdamW, an initial learning rate of 2 × 10^−5^, and weight decay of 5 × 10^−4^. A 10-epoch warmup is followed by cosine annealing to a minimum learning-rate ratio of 0.1. The classification objective is class-weighted focal loss with *γ* = 2 and label smoothing of 0.05; class weights are calculated only from retained source-training labels. For PFN–PGSA, the optimization objective additionally includes a physical-attention penalty with a coefficient of 0.3. This penalty is calculated as the squared Frobenius norm of the fused attention multiplied element-wise by one minus the harmonic mask. The mask is broadcast along the key-frequency dimension; the squared values are summed over query and key positions and averaged over samples and attention heads. EMA decay is 0.999, and gradient clipping uses a maximum norm of 1.0. Source-validation accuracy is the primary checkpoint-selection criterion, with macro-F1 used as the secondary criterion within the accuracy tolerance of 10^−4^. Early-stopping patience is 10 epochs. Only the selected checkpoint is evaluated on held-out conditions. PGMV-BAR additionally uses 30 epochs of auxiliary pretraining, as specified in Section 3.3.2; this stage is not applied to the supervised baselines.

All baselines start from random initialization without external pretrained weights. SE-ResNet convolutional weights use Kaiming normal initialization, with the final batch-normalization scale in each residual bottleneck initialized to zero. Swin backbone linear weights use a truncated normal distribution with a standard deviation of 0.02. The learned positional embeddings of PFN–PGSA are initialized with a truncated normal distribution with a standard deviation of 0.02, and its attention-mixing parameter is initialized to zero. Remaining modules use their PyTorch default initializers unless specified above. Random seeds 53, 54, and 55 control repeated runs. For BJTU-RAO, the sample-retention seed was fixed at 53 across repeated runs for all seven models. For L, the training, validation, and test retention seeds were the run seed, run seed + 1, and run seed + 2, respectively. Within each task and seed, PGMV-BAR and all six baselines used identical retained-window indices for training, validation, and testing. For L, variation across seeds therefore reflects both training randomness and changes in the sampled validation and test windows. The additional pretraining stage and hyperparameter-selection procedure of PGMV-BAR are described separately.

### 4.2. BJTU-RAO Dataset and Preprocessing

**Figure 4:**
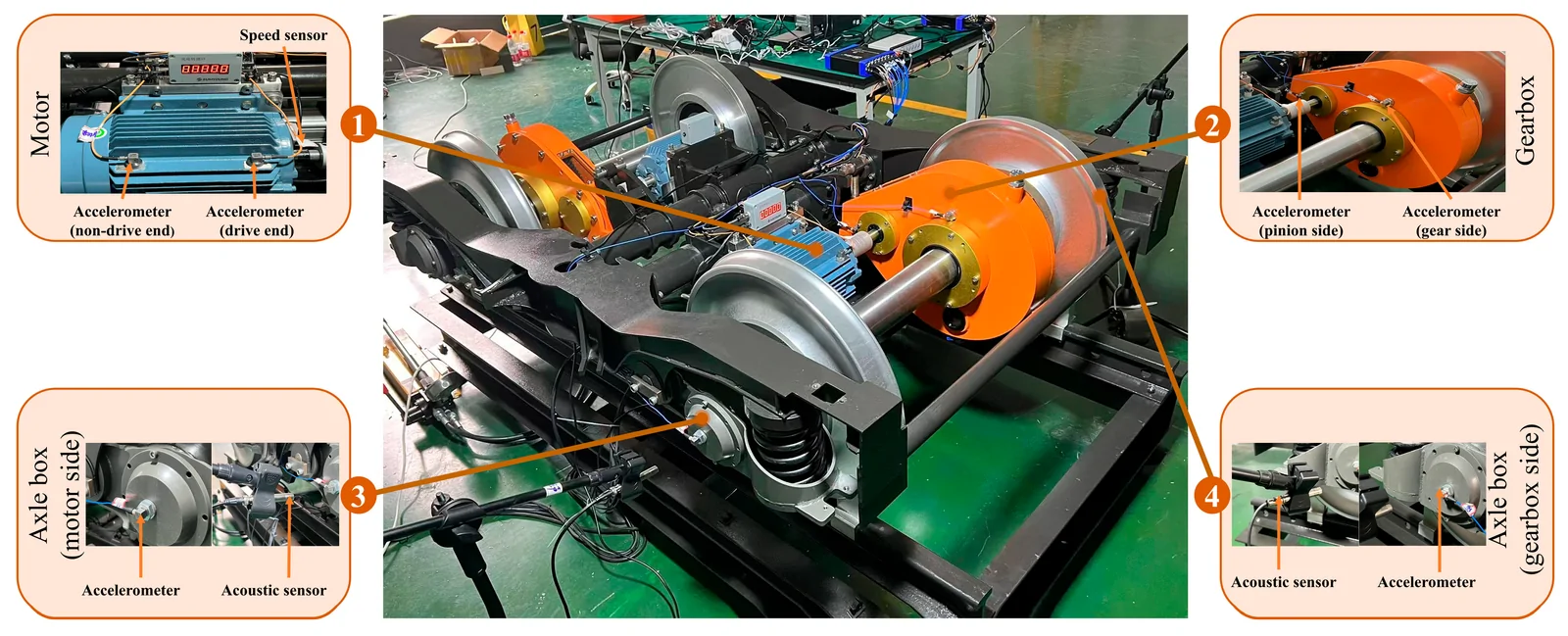
BJTU-RAO bogie transmission-system test rig.

The BJTU-RAO bogie dataset is a public dataset for fault diagnosis of railway bogie transmission systems. It was acquired from a 1:2 scaled fault-simulation test rig of a real metro-train bogie transmission system. The transmission chain consists of a traction motor, reduction gearbox, and axle box, which can reproduce vibration responses under complex railway operating environments [1]. The test rig is shown in Figure 4.

The supporting bearing of the gearbox driving gear is HRB 32305, and the axle-box bearing is HRB 352213. The gearbox adopts helical gear transmission, with 16 teeth on the pinion and 107 teeth on the gear. The bearing characteristic orders calculated using (2) are listed in Table 2. The dataset contains nine operating conditions, as listed in Table 3, and a constant vertical load of 10 kN is applied under each condition. The sampling frequency of all channels is 64 kHz, and each acquisition lasts approximately 10 s.

**Figure 5:**
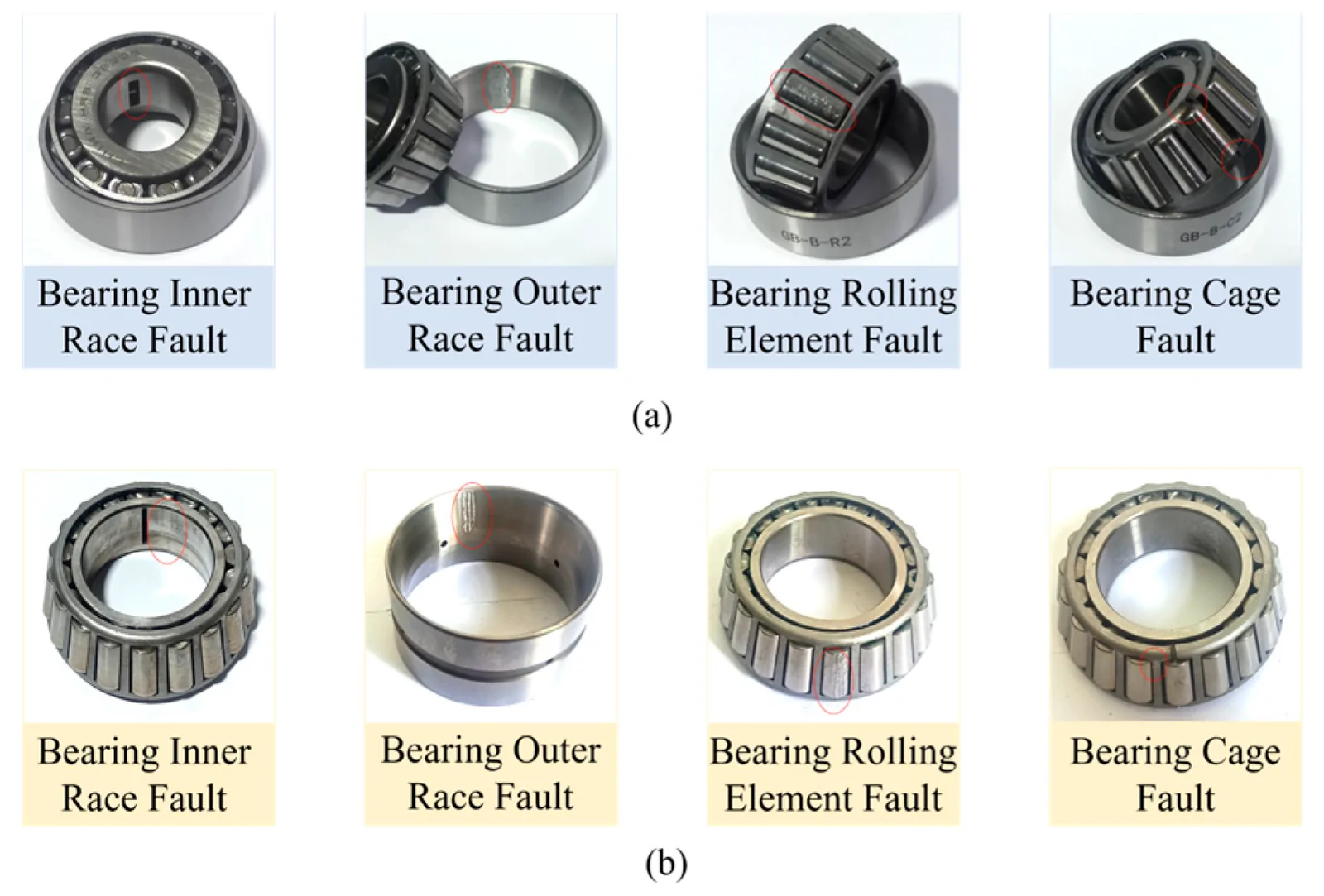
Bearing fault types in the BJTU-RAO dataset. (**a**) Typical gearbox faults. (**b**) Typical axle-box bearing faults.

Nine bearing health states were selected from the BJTU-RAO vibration recordings. The corresponding fault types are shown in Figure 5.

The sampling frequency of the raw vibration signals is 64 kHz. The sliding-window length was set to 8192 sampling points, with a stride of 2048 points, corresponding to 75% overlap between consecutive windows from the same channel. During envelope order spectrum construction, the resonance-search frequency range was set to 2–20 kHz, 12 candidate resonance bands were divided, and the order of the bandpass filter was set to 4. The maximum order of the order spectrum was set to 16, with 768 discrete sampling points. The time–frequency representation was constructed using STFT, with a window length of 512, an overlap length of 384, and a maximum retained frequency of 20 kHz. Channel selection used the topk_std strategy based on standard-deviation ranking:

For topk_std, each channel was scored by its standard deviation over its original recording, and channels were retained in descending order (three for the gearbox and two for the axle box). The selected channels were segmented separately. For resonance-band selection, the 2–20 kHz range was divided into 12 equal-width bands with boundaries at 2.0, 3.5, 5.0, 6.5, 8.0, 9.5, 11.0, 12.5, 14.0, 15.5, 17.0, 18.5, and 20.0 kHz. Each window was filtered in each candidate band using a fourth-order Butterworth filter with forward–backward filtering; the band with the largest Pearson kurtosis of its filtered waveform was selected. Band selection used the current window and did not use test labels.

Rotational-speed information was read from the operating-condition metadata. The gearbox shaft frequency was the motor frequency, whereas the axle-box shaft frequency was the motor frequency multiplied by 16/107. After Hilbert-envelope extraction and the Fourier transform, each frequency bin *f* was converted to order *f*/*f*_r_ using the appropriate shaft frequency. Linear interpolation onto 768 equally spaced order points from 0 to 16 produced the spectral vector, followed by log(1 + amplitude) compression and standardization.

For sample splitting, each BJTU-RAO task reserved two complete operating conditions for testing and used the remaining seven as source conditions. Original recordings were assigned to the source and held-out test pools by operating condition before window generation. Within each task, no original recording contributed windows to both pools. Within the source pool, segmentation preceded the training/validation split. Windows from each source recording were enumerated first by channel and then by time; approximately the first 70% formed the candidate training subset, and the remaining 30% formed source validation. Class-stratified sampling retained 20% of the candidate training windows, corresponding to approximately 14% of all source windows. The same retained subset was used for 30-epoch pretraining and subsequent supervised fine-tuning. Table 4 reports the retained signal-window counts for the BJTU-RAO task. Patch division creates tokens within each window and does not increase the number of samples.

Source training and validation were partitioned at the window level within the source recordings and can therefore contain overlapping windows or synchronous-channel observations from the same recording. Source validation was used for model selection within the source conditions. Gradient updates during pretraining and fine-tuning used only the retained source-training windows. All windows from the held-out-condition recordings were reserved for final testing and were excluded from both optimization stages and model selection. The selected checkpoint was subsequently evaluated on the two held-out conditions. Accordingly, the reported test performance characterizes cross-condition diagnosis on operating conditions that were not used for model optimization or selection.

### 4.3. L Dataset and Preprocessing

**Figure 6:**
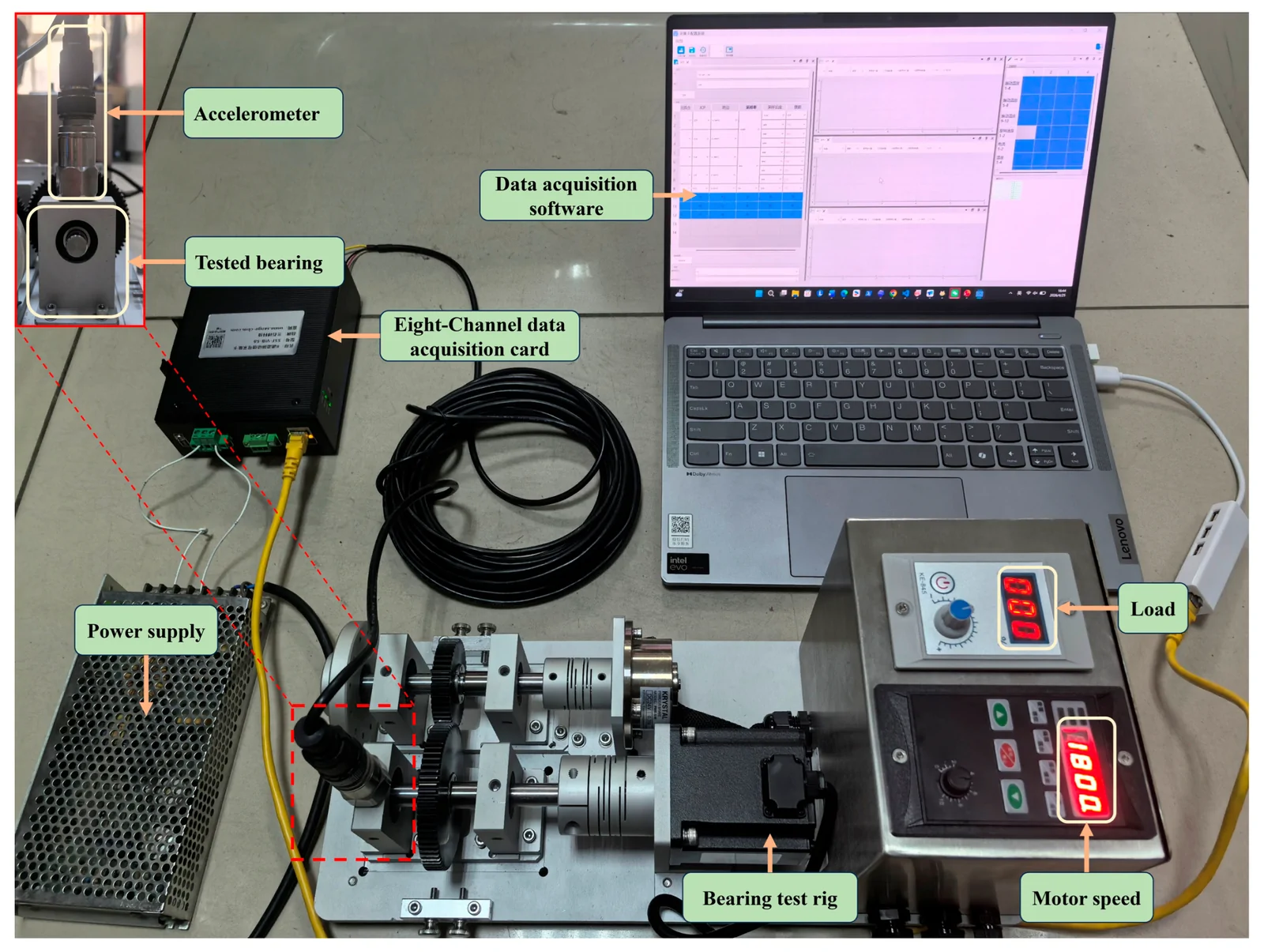
L dataset test platform.

To further verify the applicability of the proposed method in a practical acquisition scenario, a self-collected rolling bearing fault dataset, referred to as the L dataset, was constructed. The test object was an SKF 6001 deep-groove ball bearing. The experimental platform, shown in Figure 6, consists of data-acquisition software, an eight-channel data-acquisition card, a bearing test bench, acceleration sensors, and a power supply. The acquisition software, Capture Card Configuration System (CapturerConfig, version 1.2.5; Sanshifeng Technology, Tianjin, China), was deployed on the host computer for real-time monitoring, display, and storage of vibration signals. The eight-channel acquisition card was used for synchronous acquisition and transmission of sensor signals. The bearing test bench provided a controllable mechanical rotation environment. Acceleration sensors were installed at the test positions to measure vibration responses during bearing operation, and the power supply provided stable electrical power for the test system. The L dataset is publicly available on IEEE DataPort under the title “Bearing Fault L Dataset” (https://doi.org/10.21227/9r03-5q43).

During the experiment, the tested bearing was mounted on the bearing test bench, and the shaft system was driven by the drive system. Bearing vibration signals were collected under different speed and load conditions. Three rotational speeds, 1200, 1500, and 1800 r/min, were considered. For each speed, no-load and 10% load states were set, where 10% load corresponds to 10% of the rated torque of 5 N·m. Thus, six operating conditions were formed: 1200 r/min (0% rated torque), 1200 r/min (10% rated torque), 1500 r/min (0% rated torque), 1500 r/min (10% rated torque), 1800 r/min (0% rated torque), and 1800 r/min (10% rated torque), labeled C1–C6, respectively. The sampling frequency was 102.4 kHz.

For condition encoding, the rotational speeds of 1200, 1500, and 1800 r/min corresponded to normalized speed entries of 0.500, 0.625, and 0.750, respectively. The 0% and 10% rated-torque settings corresponded to normalized load entries of 0 and 1. Each load entry was duplicated to form the three-dimensional descriptor defined in Section 3.1.3. The descriptors were obtained from the operating metadata independently of the fault labels.

**Figure 7:**
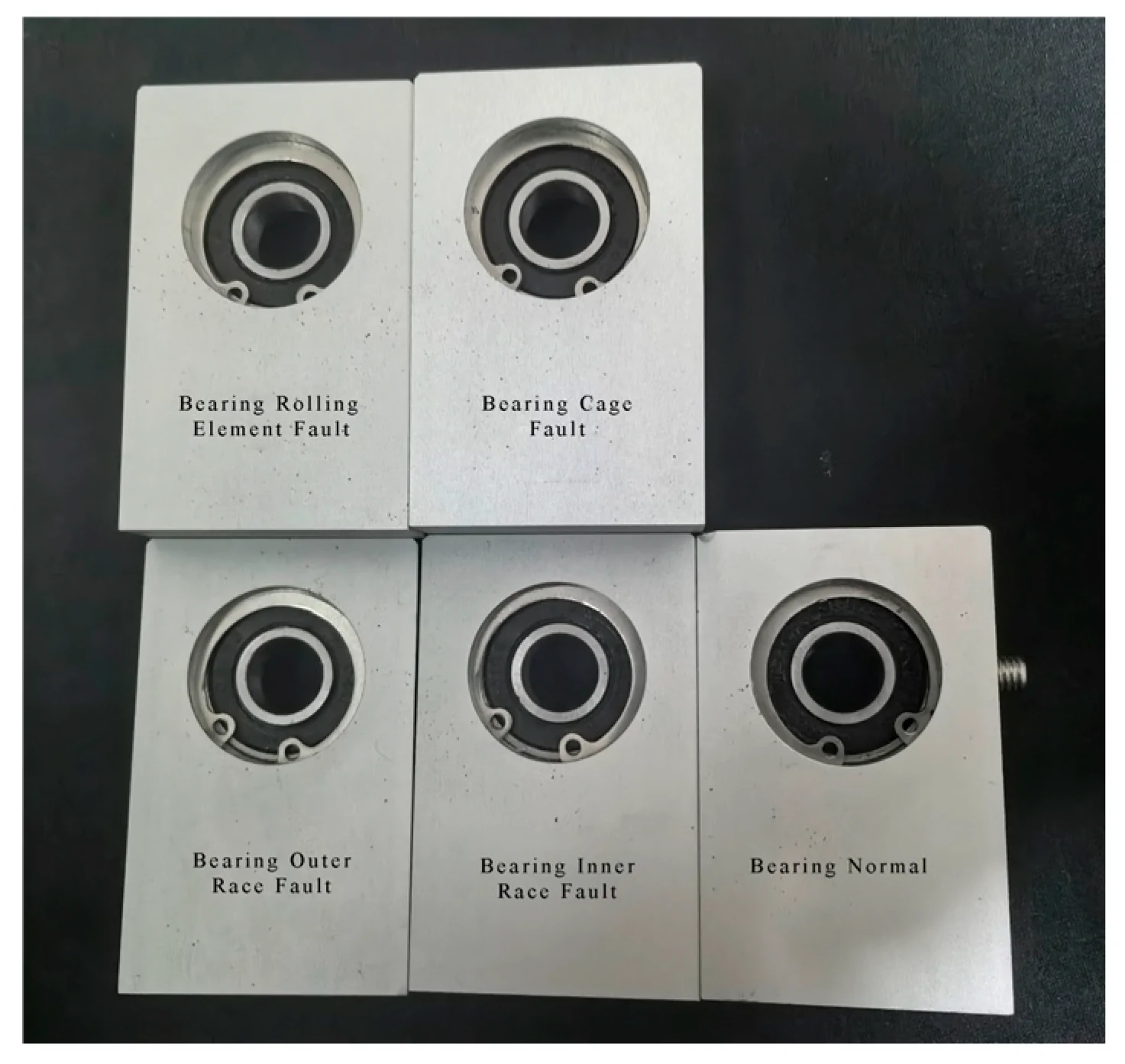
Bearing fault types in the L dataset.

The L dataset contains five bearing states: normal, inner-race fault, outer-race fault, rolling-element fault, and cage fault, as illustrated in Figure 7. Five SKF 6001 bearings were used, with one physical specimen representing each state. Each specimen was measured under all six operating conditions. According to the bearing geometry, the nominal characteristic orders of the outer-race, inner-race, cage, and rolling-element faults are approximately 3.05, 4.95, 0.38, and 1.98, respectively.

For each bearing–condition combination, one continuous acquisition was performed. Startup, shutdown, and abnormal acquisition data were excluded, after which 35 acceleration TXT records were retained, and excess records were discarded. Each retained record contained 32,768 sampling points, corresponding to 0.32 s at 102.4 kHz. The 35 records therefore represented a summed retained-signal duration of 11.2 s per combination, excluding discarded data. Across the five bearings and six conditions, the dataset comprised 30 continuous acquisition runs and 1050 retained TXT records. The 35 records within each combination originated from the same acquisition run.

A sliding-window strategy was applied separately to each 32,768-point TXT record, using an analysis-window length of 16,384 points and a stride of 4096 points. These settings correspond to 0.16 s windows, a 0.04 s step, and 75% overlap. Each record generated five windows, starting at sample indices 0, 4096, 8192, 12,288, and 16,384. All five windows were extracted directly from the stored record without padding or sampling-rate conversion.

Each analysis window was standardized separately and used to construct both observation views. For resonance-band selection, the 15–25 kHz range was divided into 10 equal-width candidate bands. Each window was filtered in each candidate band using a fourth-order Butterworth filter with forward–backward filtering, and the band yielding the largest Pearson kurtosis of the filtered waveform was selected. The selected waveform was used to construct the envelope order spectrum, represented by 384 equally spaced order points over orders 0–8. The time–frequency representation was computed using an STFT window length of 4096 points and an overlap of 3072 points. Training augmentation operated on the extracted windows while preserving their final 16,384-point length. Subsequent patch division produced tokens within each sample and did not increase the dataset sample count.

The L dataset used six leave-one-condition-out tasks. In each task, one operating condition was reserved for testing and the remaining five supplied the source data. For each class, the source pool contained 175 TXT records, and the held-out test pool contained 35 records. Condition assignment preceded window generation. All 35 retained TXT records in each bearing–condition combination were used to generate candidate windows. Each TXT file was processed as a one-dimensional acceleration record; the topk_std channel-ranking procedure used for BJTU-RAO was not applied to L.

Within each source TXT record, the five windows were ordered chronologically. Rounding the nominal validation allocation of 30% to an integer assigned two windows to candidate validation, leaving three for candidate training. The first three windows entered candidate training, and the last two entered candidate validation. Across the five source conditions, this produced 525 candidate training windows and 350 candidate validation windows per class. All 525 training windows were retained, whereas 225 validation windows were selected by seeded random sampling without replacement. The held-out condition produced 175 candidate test windows per class, from which 150 were selected without replacement. Selection was performed at the window level within each class and partition, without imposing a fixed number of retained windows per TXT record. No additional fractional training-window subsampling was applied. The final counts were therefore 525/225/150 per class, corresponding to 2625 training, 1125 validation, and 750 test windows across the five classes in each task. The retained analysis-window counts per class for each task are summarized in Table 5.

Source training and validation may contain overlapping windows from the same TXT record and correlated segments from the same continuous acquisition. Source validation was therefore used for within-source model selection rather than independent-recording evaluation. The same retained training windows were used for 30-epoch pretraining and subsequent supervised fine-tuning. Held-out-condition windows were excluded from both optimization stages and checkpoint selection. Source and held-out records belonged to different operating conditions within each task, but the same physical bearing was measured across those conditions.

## 5. Results and Discussion

### 5.1. Results on the BJTU-RAO Dataset

#### 5.1.1. Cross-Condition Diagnostic Performance and Analysis

**Figure 8:**
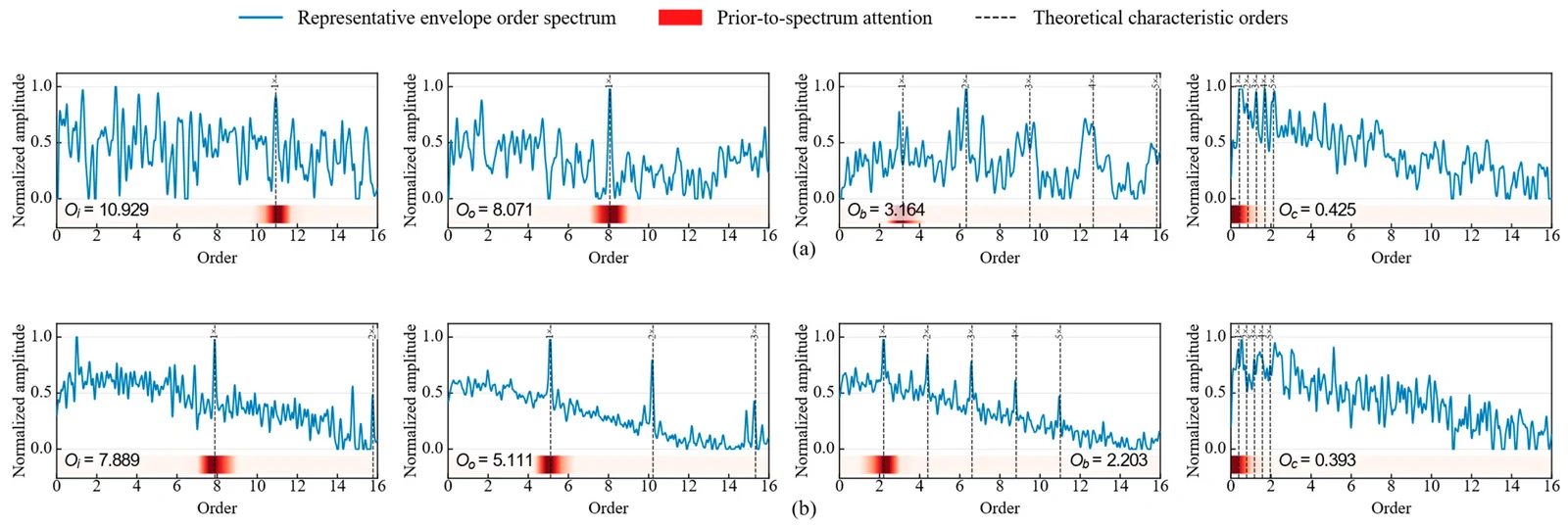
Qualitative visualization of prior-to-spectrum attention for the BJTU-RAO WC1–WC2 held-out task, with WC3–WC9 used for source training and validation: (**a**) axle-box bearings; (**b**) gearbox bearings. From left to right, the panels show inner-race, outer-race, rolling-element, and cage faults. Blue curves represent normalized envelope order spectra; black dashed lines indicate theoretical characteristic orders and the displayed harmonics; red strips show attention associated with the corresponding physical prior, with darker red indicating higher attention weights.

For the detailed BJTU-RAO task, WC3–WC9 supplied source training and validation, and WC1 (20 Hz, 0 kN lateral load) and WC2 (40 Hz, 0 kN lateral load) jointly formed the held-out test set; vertical load was 10 kN throughout. Figure 8 illustrates the relationship between the envelope order spectrum, theoretical fault orders, and prior-to-spectrum attention for axle-box and gearbox bearings. Order denotes frequency normalized by the rotational frequency of the corresponding bearing shaft. In each row, the four panels represent inner-race, outer-race, rolling-element, and cage faults, respectively. The blue curves show the normalized envelope order spectra, while the black dashed lines mark the corresponding theoretical characteristic orders and the displayed harmonics.

The attention is obtained from the cross-attention module in which physical prior tokens provide queries and encoded spectral tokens provide keys and values. With the reported configuration, the 768-point order spectrum is divided into 24 patches of 32 points each. Averaging over the eight attention heads produces a 4 × 24 attention matrix for each sample, with one row for each physical prior. The red strip in each panel visualizes the attention weights assigned by the corresponding physical prior query to the spectral patches; darker red indicates higher attention weights. This represents attention allocated to spectral patches rather than an independent importance score for every original spectral bin.

For example, the axle-box inner- and outer-race panels show concentrated attention near orders 10.929 and 8.071, respectively, where corresponding spectral peaks are visible. Similar correspondence occurs near orders 7.889 and 5.111 in the gearbox panels. These observations provide qualitative support that the prior queries allocate attention to spectral regions associated with mechanically meaningful fault periodicities. The displayed attention is mainly concentrated near the fundamental characteristic orders; the harmonic markers provide additional spectral references and do not imply equally strong attention at every harmonic.

This behavior should also be interpreted in light of the prior-alignment loss, which encourages spectral attention to follow Gaussian targets centered on the fundamental characteristic orders. Figure 8 therefore illustrates the behavior of the regularized attention mechanism.

To complement the qualitative visualization, a fixed-checkpoint sensitivity analysis was conducted on the same BJTU-RAO WC1–WC2 held-out task using 14,880 test windows. The four characteristic-order priors were jointly perturbed by −10%, −5%, +5%, and +10%, while model weights, observation inputs, and operating-condition descriptors remained fixed. Relative to the unperturbed head-averaged spectral attention, the mean Jensen–Shannon divergences were 0.010015, 0.001925, 0.001571, and 0.008665 nats, respectively, with corresponding mean absolute attention-centroid displacements of 0.050326, 0.024164, 0.021144, and 0.057445 order units. These measures were averaged over the four prior queries and all test windows. Accuracy and macro-F1 remained 99.1263% and 99.1356%, respectively, across all tested perturbations. The results quantitatively demonstrate that the attention distribution responds to the supplied characteristic-order values, with larger mean changes at ±10% than at the corresponding ±5% perturbations, while classification performance remains stable within this range.

In addition, class-wise recall *R_x_* was used to measure the recognition capability for different fault types and further characterize the diagnostic performance of difficult classes under cross-condition scenarios. Under this setting, each metric was calculated on the test set, and the results are reported in Table 6.

The proposed method achieved 99.13 ± 0.04% accuracy on the test set, with macro-precision, macro-recall, and macro-F1 values of 99.21%, 99.05%, and 99.13%, respectively. These results indicate high overall diagnostic accuracy and balanced class-wise recognition in the two-condition test task. From the class-wise recall results, most classes approached or reached 100%, with only slight decreases in a few classes, demonstrating stable recognition for most fault types.

**Figure 9:**
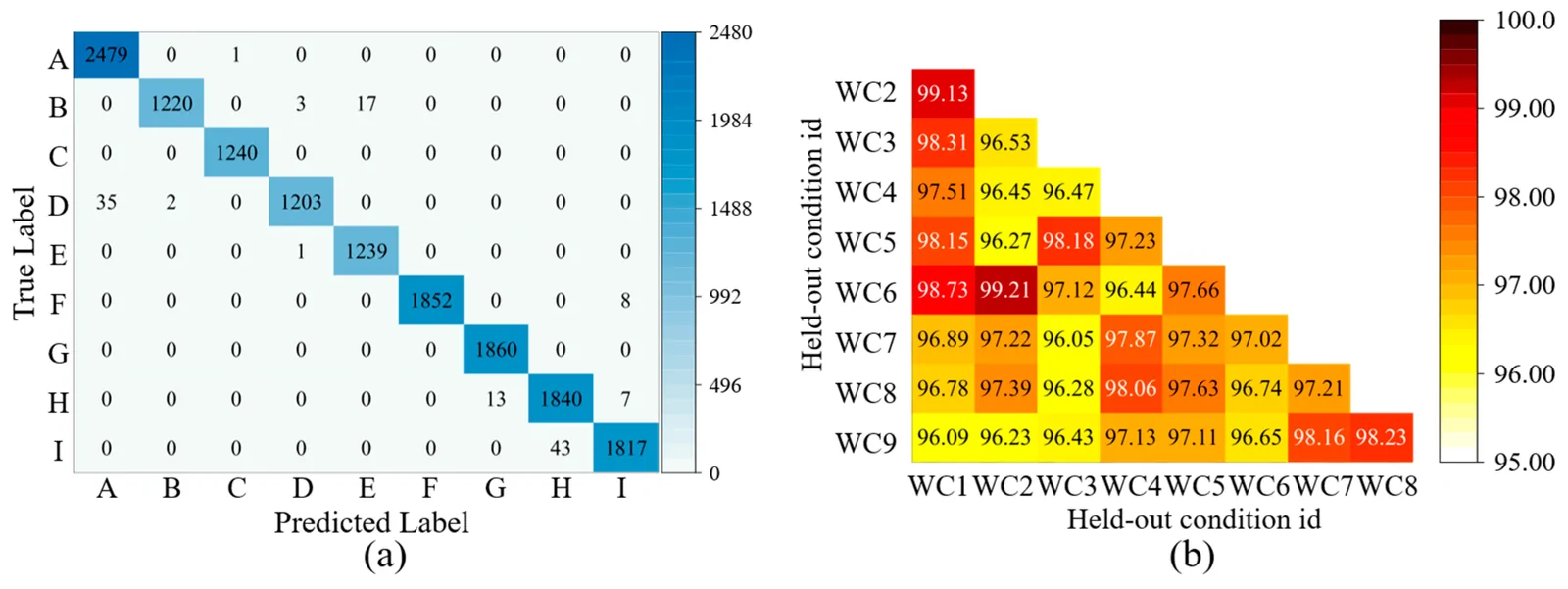
Cross-condition diagnostic performance on BJTU-RAO. (**a**) Mean confusion matrix for the WC1–WC2 held-out task. (**b**) Mean test accuracy across the same three seeds for all 36 leave-two-conditions-out tasks.

Figure 9a presents the mean confusion matrix across seeds 53, 54, and 55 for the WC1–WC2 held-out task. Predictions are concentrated along the main diagonal, with classes C and G correctly identified in the displayed matrix. Most other classes also show limited misclassification, while the remaining errors are concentrated in a small number of class pairs.

Extended training and testing covered all 36 unordered pairs of held-out BJTU-RAO conditions. For each task, the other seven conditions provided source training and validation; pretraining and fine-tuning used only the retained source-training windows, and source validation selected the checkpoint. Figure 9b summarizes the mean test accuracy across the three runs for each held-out condition pair.

Across the 36 tasks, mean test accuracy ranges from 96.05% to 99.21%, with an overall average of 97.27%. Most condition pairs achieve mean accuracies above 97%, showing consistently high diagnostic performance across the evaluated speed–load combinations. Figure 9 therefore presents complementary evidence at two levels: class-wise recognition within the detailed WC1–WC2 task and diagnostic performance across the complete set of held-out condition pairs.

**Figure 10:**
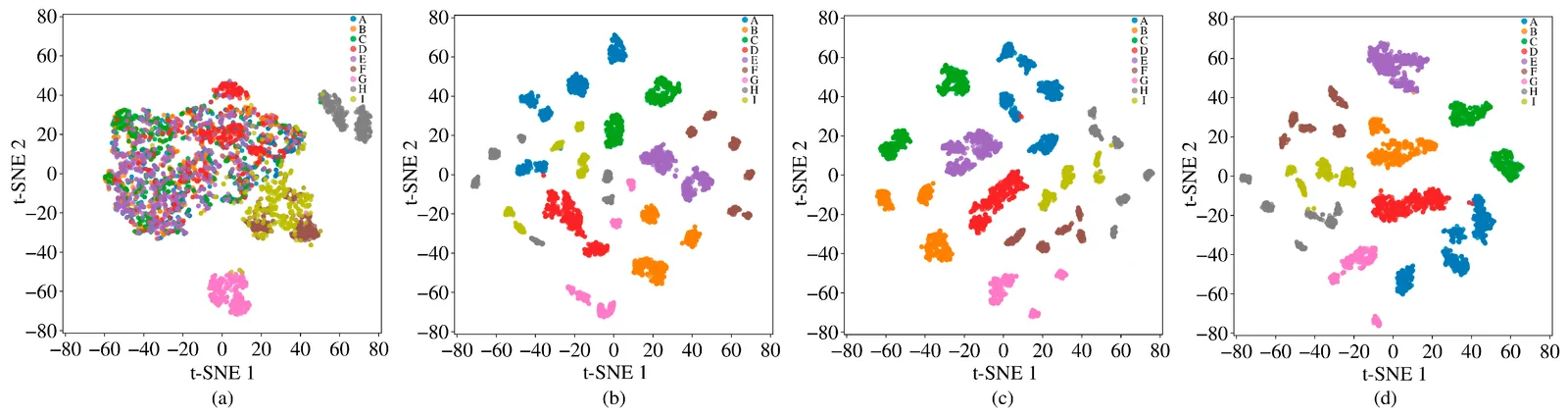
t-SNE visualizations of learned representations for the BJTU-RAO WC1–WC2 held-out task. (**a**) Spectral-view representation. (**b**) Time–frequency-view representation. (**c**) Fused representation. (**d**) Classifier output.

Figure 10 presents t-SNE visualizations of the spectral-view, time–frequency-view, fused, and classifier-output representations for the BJTU-RAO WC1–WC2 held-out task. The spectral and time–frequency representations are obtained from parallel observation branches. The spectral representation exhibits substantial interclass overlap, whereas the time–frequency representation shows more distinct class clusters. The fused and classifier-output representations also exhibit class-related clustering, although some classes form multiple groups. These visualizations provide qualitative evidence of the learned representations and complement the quantitative test results in Table 6.

Together, the classification results and feature visualizations provide complementary evidence of cross-condition diagnostic performance and the discriminative representations learned by PGMV-BAR within the evaluated BJTU-RAO setting.

#### 5.1.2. Baseline Comparison

PGMV-BAR and six implemented baselines were evaluated across all 36 BJTU-RAO leave-two-conditions-out tasks. All methods followed the experimental protocols described in Section 4.1. Table 7 summarizes their overall accuracies across the complete set of held-out condition pairs.

PGMV-BAR achieved the highest overall BJTU-RAO accuracy of 97.27 ± 0.14%, compared with 92.54 ± 0.13% for TF-ResNet, the strongest baseline by overall mean accuracy. The paired improvement was 4.73 ± 0.21 percentage points across seeds. PGMV-BAR ranked first on 34 of the 36 tasks and exceeded TF-ResNet on 35 tasks. The source-only PFN–PGSA implementation achieved 38.65 ± 0.05%. These complete-task results characterize performance across the full set of evaluated speed–load combinations.

#### 5.1.3. Ablation Results

Table 8 summarizes the component and training-strategy ablations, computational profiles, and numerical-prior controls on the BJTU-RAO WC1–WC2 task. The architectural variants used 30 pretraining epochs followed by at most 100 fine-tuning epochs under the common data-partition and training protocol. The no-pretraining variant retained the complete architecture and the same fine-tuning objective, source-training windows, optimization settings, and checkpoint-selection criteria, while omitting the auxiliary pretraining stage. For the BAR comparison, all three configurations retained 16 fusion layers and shared the maximum training budgets and stopping criteria. Their parameter counts and computational costs are reported in Table 8b.

From the input-view perspective, Spec-Only, Time-Only, and TF-Only achieved accuracies of 64.36%, 78.79%, and 94.21%, respectively. The complete model exceeded these variants by 34.77, 20.34, and 4.92 percentage points, supporting the complementary contribution of the selected spectral and time–frequency views. Adding a raw-time branch yielded 93.92% accuracy, below the complete model, indicating that an additional observation view did not automatically improve performance on this task.

For the BAR comparison, the eight-block, four-block, and no-BAR configurations achieved accuracies of 99.13 ± 0.04%, 95.43 ± 0.19%, and 94.81 ± 0.34%, respectively. The eight-block and four-block configurations had identical fusion depth and trainable parameter counts, while their estimated computational costs were close at 14.188 and 14.172 GFLOPs per window. Their 3.70-percentage-point accuracy difference therefore supports the selected BAR grouping beyond an increase in parameter count or fusion depth. Compared with the no-BAR variant, the complete model added only 25,344 trainable parameters, approximately 0.047%, and achieved a 4.32-percentage-point accuracy improvement. These results characterize the evaluated configurations under identical or closely matched parameter counts and the shared training protocol.

The complete model required an estimated 14.188 GFLOPs per window and 225.26 MiB of peak allocated GPU memory. Its network latency was 10.89 ± 0.49 ms, compared with 6.23 ± 0.32 ms without BAR. Thus, the approximately 0.3% increase in estimated arithmetic operations was accompanied by a measurable 4.66 ms increase in network latency. Including preprocessing, data transfer, and prediction return, the complete model required 22.04 ± 0.29 ms per window, with a P95 latency of 22.20 ms and a maximum observed latency of 25.20 ms.

Ablations related to physical priors support the usefulness of the interaction and regularization pathways within the evaluated framework. Removing prior cross-attention, prior alignment, or prior tokens reduced accuracy from 99.13% to 94.48%, 92.22%, and 92.94%, respectively, corresponding to decreases of 4.65, 6.91, and 6.19 percentage points.

To examine the role of the numerical priors while retaining the complete architecture, three additional configurations were trained using the common protocol: a uniform +20% shift of the nominal orders, a fixed cyclic permutation of the four orders, and location-specific random orders sampled uniformly from [0, 16). The random table remained fixed across samples and training seeds. Each configuration supplied the same controlled orders to the prior-token encoder and the alignment target throughout training and evaluation. All configurations used seeds 53, 54, and 55, with 30 pretraining epochs followed by at most 100 fine-tuning epochs.

As shown in Table 8c, nominal priors achieved 99.13 ± 0.04% accuracy, exceeding the shifted, permuted, and random-prior configurations by 3.24, 2.60, and 4.49 percentage points, respectively. The corresponding macro-F1 decreases were 4.00, 2.97, and 5.79 percentage points. These comparisons support the contribution of nominal characteristic-order information to the joint prior-query and alignment mechanism under the same architecture and training protocol. The permutation control examines query-slot assignment while preserving the nominal order values; the shifted and random controls directly test altered numerical priors.

Removing the entire condition token reduced accuracy from 99.13 ± 0.04% to 94.65 ± 0.68% and macro-F1 from 99.13 ± 0.02% to 93.65 ± 0.61%, corresponding to mean decreases of 4.48 and 5.48 percentage points, respectively. This ablation removed the continuous speed/load encoding, bearing-location embedding, and condition-token-type embedding from the fusion sequence. The observation branches, physical-prior pathway, and BAR fusion architecture were retained. Rotational-speed information remained available for order-spectrum construction. These results support the contribution of the complete explicit conditioning pathway on this task.

Removing auxiliary pretraining reduced accuracy from 99.13 ± 0.04% to 96.30 ± 0.27% and macro-F1 from 99.13 ± 0.02% to 95.77 ± 0.21%. The ablated model retained the complete architecture and was trained directly using the fine-tuning objective. The 2.83-percentage-point accuracy decrease supports the benefit of adding the 30-epoch auxiliary pretraining stage under the same supervised-stage training settings.

Regarding weak-class recognition, the full model achieved a worst-class recall of 97.02%, compared with 23.87% for Spec-Only and 43.47% for Time-Only.

Taken together, the ablations support the contributions of the complementary observation views, physical-prior pathways, explicit conditioning, BAR-based fusion, and auxiliary pretraining within the evaluated framework. The BAR comparisons document the corresponding parameter and depth controls, while the no-pretraining comparison quantifies the benefit of including the auxiliary stage under the same fine-tuning settings. The PFN–PGSA comparisons in Section 5.1.2 and Section 5.2.2 provide complementary evidence at the complete-framework level.

### 5.2. Results on the L Dataset

#### 5.2.1. Leave-One-Condition-Out Results and Analysis

Table 9 reports the leave-one-condition-out test results under the six operating conditions.

The proposed method obtains average accuracy, macro-precision, macro-recall, and macro-F1 values of 95.33 ± 0.58%, 95.53%, 95.33%, and 95.33%, respectively, indicating good cross-condition generalization to unseen operating conditions.

Across conditions, C1, C3, and C4 show better recognition performance, with accuracies of 97.73%, 97.20%, and 96.80%, respectively. In contrast, C5 and C6 are more challenging, with accuracies decreasing to 92.00% and 93.07% and macro-F1 values of 91.99% and 93.08%, respectively. This indicates more evident distribution discrepancies between these two test conditions and the training conditions.

For class-wise recall, fault type 2 achieves the highest average recall of 99.22%, indicating stable recognition across operating conditions. Fault types 1, 4, and 5 also maintain average recall values of 95.67%, 94.67%, and 95.00%, respectively. In contrast, fault type 3 has the lowest average recall of 92.11%, mainly because its recall decreases to 84.00% and 83.33% under C5 and C6, respectively. This suggests that fault type 3 is more sensitive to operating-condition variations and is more prone to missed detection or misclassification under complex conditions.

To investigate the outer-race errors, supplementary analyses used fixed checkpoints trained with seeds 53 and 54 for C5 and C6, respectively, separately from the three-seed summaries in Table 9. Each analysis included 150 outer-race windows, of which 127 in C5 and 121 in C6 were classified correctly. The target band was centered at order 3.05 with a half-width of 0.35 order units. Within the range 0 < order ≤ 8, the background comprised all bins outside the target band. Spectral contrast was expressed in decibels as 10 times the base-10 logarithm of the ratio of mean target-band power to mean background power.

In C5, the median contrast was 5.34 dB for correctly classified windows and 0.48 dB for incorrectly classified windows, while the corresponding median raw RMS values were 1.305 and 7.806 recorded-signal units. These errors were associated with reduced relative prominence of the nominal outer-race band despite higher overall vibration amplitude. In C6, the median contrasts were similar, at 5.58 and 5.62 dB, and 25 of the 29 incorrect outer-race predictions were assigned to cage faults. The two conditions therefore exhibited different error patterns. Fault severity and sensor location were not independently varied, so these analyses describe associations within the available acquisition setting.

Spectral-patch occlusion compared high-attention, low-attention, and random selections using zero and spectral-median replacement, with model weights and other inputs held fixed. One, two, or four of the 12 spectral patches were replaced, corresponding to masking fractions of 8.33%, 16.67%, and 33.33%; five random-mask repetitions were averaged within each window. The target was each window’s original predicted class, held fixed across interventions. Normalized AOPC was calculated by trapezoidal integration of the target-probability-drop curve, including the unoccluded origin, and division by the maximum masking fraction of one third. C5 showed negligible probability changes. For initially correct C6 outer-race windows, high-attention normalized AOPC was 0.017784 and 0.020747 under the two replacement rules, compared with 0.057023 and 0.057250 for random masking. These results show prediction sensitivity to spectral removal in C6, but do not establish attention magnitude as a ranking of classification contribution. The attention visualizations are therefore interpreted as physically referenced information allocation, with prediction relevance assessed separately through controlled interventions.

**Figure 11:**
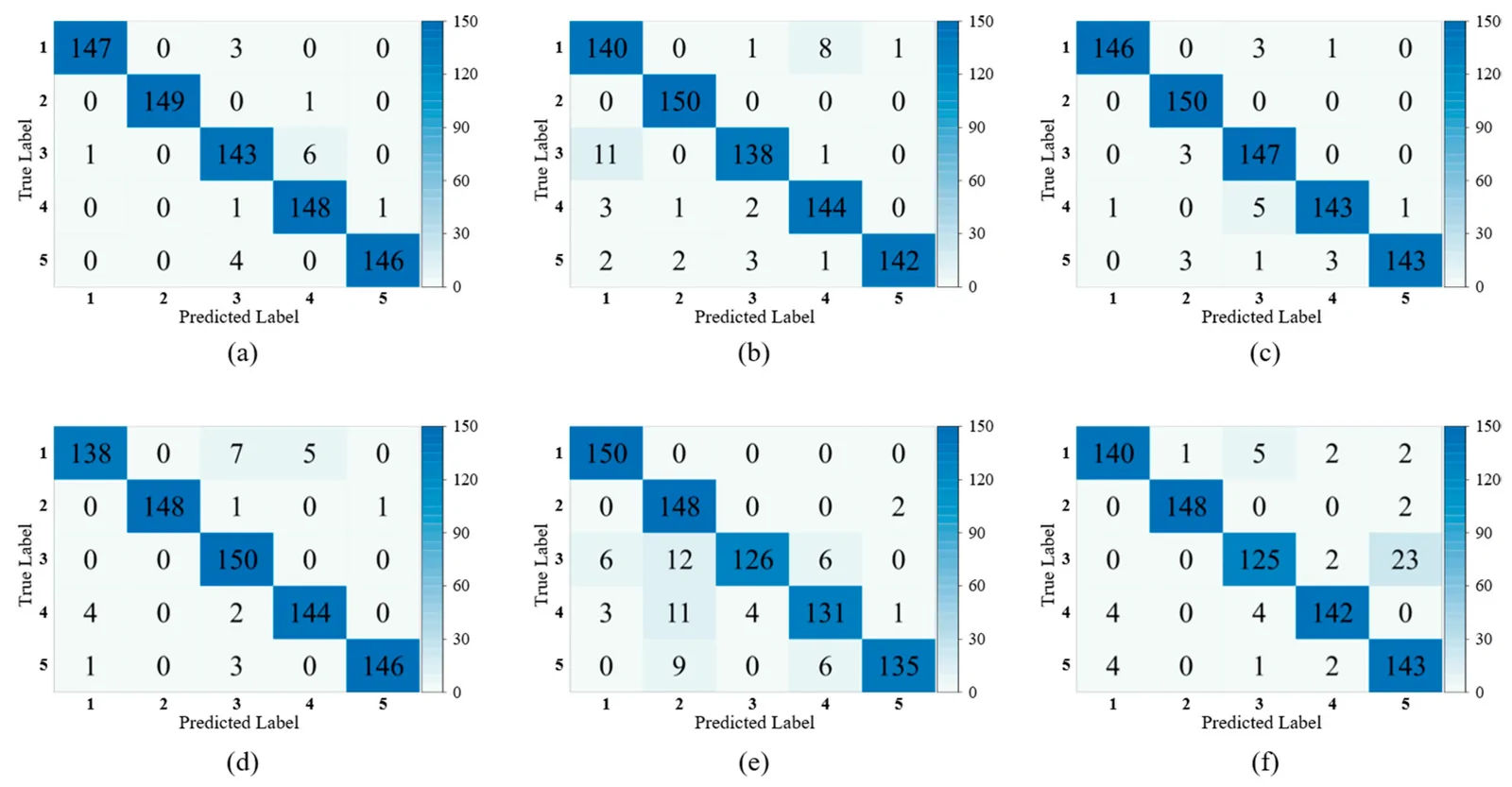
Mean confusion matrices for the six L-dataset leave-one-condition-out tasks, obtained by elementwise averaging across seeds 53, 54, and 55. (**a**) C1. (**b**) C2. (**c**) C3. (**d**) C4. (**e**) C5. (**f**) C6.

Figure 11 presents the mean confusion matrices across seeds 53, 54, and 55 for the six leave-one-condition-out tasks, complementing the checkpoint-specific error analyses reported above.

The confusion matrices show that most predictions are distributed along the main diagonal under all conditions, indicating stable recognition capability under unseen operating conditions.

**Figure 12:**
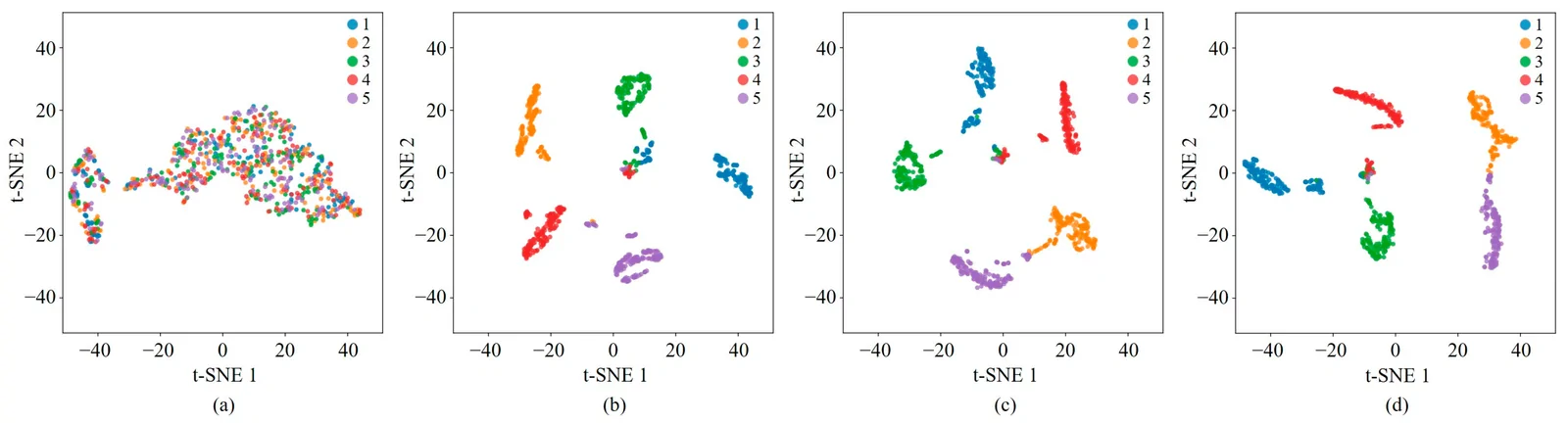
t-SNE visualization for the L-dataset C1 held-out task. (**a**) Spectral view. (**b**) Time–frequency view. (**c**) Fusion feature. (**d**) Classifier output.

Figure 12 presents t-SNE visualizations for the C1 held-out task on the L dataset. These visualizations provide qualitative evidence of the discriminative representations learned by PGMV-BAR and complement the quantitative classification results for this held-out task.

#### 5.2.2. Cross-Condition Comparison with Baseline Models

Table 10 summarizes the overall accuracies of PGMV-BAR and the six implemented baselines across the six L-dataset leave-one-condition-out tasks. The implementations follow Section 4.1, with the dataset-specific preprocessing and evaluation protocol described in Section 4.3.

Across the six L-dataset holdouts, PGMV-BAR achieved the highest overall accuracy of 95.33 ± 0.58%, compared with 87.20 ± 0.54% for TF-Swin-Transformer, the strongest baseline by overall mean accuracy. The paired improvement was 8.13 ± 0.68 percentage points across seeds. PGMV-BAR obtained higher three-seed mean accuracy than all six baselines under every held-out condition. The source-only PFN–PGSA implementation achieved 34.30 ± 2.81%, corresponding to a paired difference of 61.03 ± 2.32 percentage points. These comparisons characterize the implemented models under the specified source-only evaluation protocol.

Condition-wise, PGMV-BAR exceeded the strongest baseline by 0.93–14.80 percentage points. The largest margins occurred at C5 and C6, corresponding to 1800 r/min at 0% and 10% rated torque. PGMV-BAR achieved accuracies of 92.00% and 93.07%, compared with 77.73% for TF-ResNet and 78.27% for TF-Swin-Transformer, yielding improvements of 14.27 and 14.80 percentage points, respectively. These results show that the framework retained an advantage under the two highest-speed operating conditions. The class-wise results in Table 9 identify outer-race recognition as a remaining focus for refinement, with recalls of 84.00% and 83.33% under C5 and C6.

Across both datasets, the aggregate accuracy differences favored PGMV-BAR in every seed. The exact paired tests yielded unadjusted *p*-values of 0.25 and Holm-adjusted values of 1.00. The comparisons are therefore interpreted through their observed effect sizes and task-wise consistency without claiming statistical significance under this three-seed analysis.

### 5.3. Broader Implications and Limitations

The broader relevance of PGMV-BAR lies in assigning physical knowledge an explicit computational role. Xie et al. [29] combined mechanics-based modeling of recoverable deformation with Bayesian optimization for the constrained design of auxetic cementitious energy harvesters. Their approach incorporated physical mechanisms into objective evaluation and design search, whereas PGMV-BAR incorporates nominal bearing orders into query representations and attention regularization. These complementary examples illustrate how physically meaningful information can guide learning and engineering decision-making across different intelligent monitoring and optimization tasks.

The evaluations establish cross-condition performance within the bearing configurations, operating ranges, and acquisition settings represented by BJTU-RAO and L. The two datasets were evaluated through separate training and testing protocols, providing complementary evidence from different experimental platforms. Within each task, the test conditions were excluded from model optimization and selection. For L, the same bearing specimens were measured across operating conditions. Evaluation using additional specimens and independently acquired runs would broaden the assessment of performance variability. Applying a trained model directly to another machine or acquisition system represents a further stage of cross-platform validation.

The physical guidance uses nominal characteristic orders and operating descriptors as reference information. Changes in bearing geometry require recalculation of the characteristic orders using the corresponding kinematic parameters and shaft-speed reference. Resonance-band selection also depends on the measurement setting: the present implementation selects a band for each window within dataset-specific search ranges of 2–20 kHz for BJTU-RAO and 15–25 kHz for L. Changes in rotational-speed range, loading, sensor location, vibration transmission path, or acquisition system may require reassessment of these search ranges and associated preprocessing settings. Frequency ranges should remain compatible with the sampling rate and measured resonance response. Order normalization provides a common reference for nominal fault periodicities, while slip, speed-estimation error, and within-window speed variation motivate uncertainty-aware prior representations and adaptive preprocessing. The class-wise results also identify high-speed outer-race diagnosis as a focused direction for further refinement. The matched prior-control experiments further support the contribution of nominal characteristic-order information to the joint prior-query and alignment mechanism on the evaluated BJTU-RAO task.

The present diagnostic task considers classification among predefined bearing health states. Extending the framework to unknown faults, compound faults, and progressive degradation would broaden its contribution to maintenance decision-making. Such extensions could combine the proposed representation-learning architecture with unknown-fault detection and temporal health assessment.

For continuous railway monitoring, the measured processing cost provides a basis for system integration. The BJTU-RAO stride of 2048 samples at 64 kHz corresponds to a 32 ms update interval. The complete model’s processing-pipeline latency averaged 22.04 ms, with a P95 of 22.20 ms and a maximum of 25.20 ms across 150 measurements. All measured processing times were therefore below the update interval, supporting compatibility with the specified window update rate on the evaluated workstation. Validation on representative onboard hardware under sustained acquisition and processing workloads remains a subsequent step toward real-time railway deployment.

## 6. Conclusions

This study proposed PGMV-BAR for cross-condition railway bearing fault diagnosis. Characteristic-order prior tokens first queried envelope-order spectral features, and the updated representations subsequently queried time–frequency features. The resulting prior representations were retained alongside both observation streams and a separate condition token for joint fusion. Spectral prior alignment provided a physical reference for the interaction, while BAR supported information aggregation across fusion blocks. The framework was trained through auxiliary pretraining followed by supervised fine-tuning.

PGMV-BAR achieved the highest overall mean accuracy among the seven evaluated models, reaching 97.27 ± 0.14% across all 36 BJTU-RAO tasks and 95.33 ± 0.58% across the six L-dataset holdouts. It ranked first on 34 BJTU-RAO tasks and all six L tasks. The mean improvements over the strongest baselines were 4.73 and 8.13 percentage points, respectively. Component and training-strategy ablations supported the contributions of the evaluated pathways and auxiliary pretraining. Matched prior-control experiments on the BJTU-RAO WC1–WC2 task further supported the contribution of nominal characteristic-order information to the joint prior-query and alignment mechanism.

Overall, this study provided an experimentally evaluated approach to integrating bearing-kinematic information with multi-view feature learning. The evaluations established cross-condition performance within the respective dataset settings. Future work will focus on transfer across machines and acquisition systems, uncertainty-aware physical guidance, broader fault categories, and continuous monitoring under field conditions.

## Figures and Tables

**Table 1:** Hyperparameter Settings of the Proposed Method.

Category	Parameter	Value	Category	Parameter	Value
Model architecture	Embedding dimension	384	Training	Batch size	16
	Number of attention heads	8		Pretraining epochs	30
	Branch depth	6		Max fine-tuning epochs	100
	Fusion depth	16		Activation	GELU
	MLP expansion ratio	4.0		EMA decay	0.999
	Dropout rate	0.15		Grad. clipping	1.0
Patch setting	Spectral patch length	32	Learning rate schedule	Warmup epochs	10
	TF patch size	8 × 8		Min. LR ratio	0.1
	Projection dimension	64		Scheduling strategy	Warmup + cosine annealing
BAR	Branch BAR	Not used	Early stopping	Primary metric	Accuracy
	Fusion BAR	Used		Secondary metric	Macro-F1
	BAR block size	2 layers		Patience	10
	Number of BAR blocks	8		Min. improvement	1 × 10^−4^
Optimization	Optimizer	AdamW	Classification loss	Loss	Focal Loss
	Initial learning rate	2 × 10^−5^		Focal gamma	2.0
	Weight decay	5 × 10^−4^		Label smoothing	0.05
Auxiliary setting	Masking ratio	0.1	Physical prior	Prior-align. weight	0.1
	Masked loss weight	0.1	Reference condition	Reference speed	40 Hz
	Contrastive loss weight	0.05		Reference vertical load	10 kN
	Temperature coefficient	0.2		Reference lateral load	10 kN

The vertical- and lateral-load references apply to BJTU-RAO. For L, condition encoding uses a speed reference of 40 Hz and a torque-fraction reference of 0.1, with the normalized torque entry duplicated as defined in Section 3.1.3.

**Table 2:** Characteristic Orders of the BJTU-RAO Bearings.

Bearing Model	*O*_o_	*O*_i_	*O*_c_	*O*_b_
HRB 32305 (gearbox)	5.111	7.889	0.393	2.203
HRB 352213 (axle box)	8.071	10.929	0.425	3.164

**Table 3:** Operating Conditions of the BJTU-RAO Dataset.

Condition	Motor Rotational Frequency	Lateral Load	Condition	Motor Rotational Frequency	Lateral Load
WC1	20 Hz	0 kN	WC6	60 Hz	+10 kN
WC2	40 Hz	0 kN	WC7	20 Hz	−10 kN
WC3	60 Hz	0 kN	WC8	40 Hz	−10 kN
WC4	20 Hz	+10 kN	WC9	60 Hz	−10 kN
WC5	40 Hz	+10 kN	-	-	-

**Table 4:** Retained signal-window counts for the BJTU-RAO task trained on WC3–WC9 and tested jointly on WC1–WC2.

Bearing Location	Bearing Fault Type	Label	Samples
			Training/Validation/Test
Axle box (left and right)	Normal	A	1215/2604/2480
Axle box (left)	Inner-race fault	B	608/1302/1240
	Outer-race fault	C	608/1302/1240
	Rolling-element fault	D	608/1302/1240
	Cage fault	E	608/1302/1240
gearbox	Inner-race fault	F	911/1953/1860
	Outer-race fault	G	911/1953/1860
	Rolling-element fault	H	911/1953/1860
	Cage fault	I	911/1953/1860

**Table 5:** Retained analysis-window counts per class for each L-dataset leave-one-condition-out task.

Bearing Fault Type	Label	Samples
		Training/Validation/Test
Normal	1	525/225/150
Inner-race fault	2	525/225/150
Outer-race fault	3	525/225/150
Rolling-element fault	4	525/225/150
Cage fault	5	525/225/150

The reported counts represent retained analysis windows, not independent acquisition runs, physical bearing specimens, or Transformer tokens.

**Table 6:** Diagnostic Results on the BJTU-RAO WC1-WC2 Task.

Metric	Value (%)	Metric	Value (%)	Metric	Value (%)
Accuracy	99.13 ± 0.04	*R*_B_	98.39 ± 0.42	*R*_G_	100.00 ± 0.00
Macro-Precision	99.21 ± 0.03	*R*_C_	100.00 ± 0.00	*R*_H_	98.92 ± 0.11
Macro-Recall	99.05 ± 0.02	*R*_D_	97.02 ± 0.37	*R*_I_	97.69 ± 0.32
Macro-F1	99.13 ± 0.02	*R*_E_	99.92 ± 0.08	—	—
*R*_A_	99.96 ± 0.04	*R*_F_	99.57 ± 0.16	—	—

**Table 7:** Overall Accuracy Across All 36 BJTU-RAO Held-Out Condition Pairs.

Model	Accuracy (%)
PGMV-BAR	97.27 ± 0.14
1D-CNN [25]	89.22 ± 0.06
MpResCNN-BiLSTM-Attention [2]	87.92 ± 0.51
TF-ResNet [26]	92.54 ± 0.13
TF-Swin-Transformer [27]	92.11 ± 0.10
Spec+TF Dual-Branch Fusion [3,28]	86.83 ± 0.60
PFN–PGSA [16]	38.65 ± 0.05

**Table 8:** Ablation results, computational profiles, and prior-control comparisons on BJTU-RAO: (**a**) diagnostic performance on the WC1–WC2 task; (**b**) computational cost of the BAR configurations; (**c**) matched numerical-prior controls on the WC1–WC2 task.

**(a) Diagnostic Performance on the WC1–WC2 Task**								
**Variant**		**Accuracy (%)**	**Macro-Precision** **(%)**	**Macro-Recall** **(%)**	**Macro-F1 (%)**	**Early Stopping Epoch**	**Best Val. Accuracy (%)**	**Worst Class/Recall (%)**
PGMV-BAR		99.13 ± 0.04	99.21 ± 0.03	99.05 ± 0.02	99.13 ± 0.02	46.33 ± 3.79	99.58 ± 0.40	D/97.02 ± 0.37
Spec-TF Fusion with 4 BAR Blocks		95.43 ± 0.19	95.56 ± 0.18	94.28 ± 0.14	94.57 ± 0.16	40.33 ± 3.21	99.23 ± 0.11	C/70.24 ± 0.78
Spec-TF Fusion w/o BAR		94.81 ± 0.34	96.11 ± 0.39	93.93 ± 0.38	94.65 ± 0.40	35.00 ± 4.36	99.17 ± 0.57	C/72.58 ± 0.99
Time-Spec-TF Fusion		93.92 ± 0.03	94.44 ± 0.03	93.56 ± 0.03	93.79 ± 0.03	56.67 ± 3.51	99.41 ± 0.05	C/77.55 ± 0.47
Time-Spec Fusion		89.61 ± 0.18	89.03 ± 0.20	86.92 ± 0.19	87.04 ± 0.19	87.00 ± 4.58	93.93 ± 0.11	C/45.30 ± 0.82
Time-TF Fusion		91.48 ± 0.27	92.93 ± 0.18	90.99 ± 0.30	91.06 ± 0.26	73.00 ± 3.00	99.62 ± 0.18	H/61.61 ± 1.56
Time-Only		78.79 ± 0.05	79.46 ± 0.06	76.62 ± 0.05	76.76 ± 0.06	70.67 ± 1.53	81.43 ± 0.29	C/43.47 ± 2.40
Spec-Only		64.36 ± 0.17	61.66 ± 0.17	60.85 ± 0.16	60.53 ± 0.16	49.00 ± 4.00	71.35 ± 0.46	B/23.87 ± 1.34
TF-Only		94.21 ± 0.17	93.73 ± 0.17	94.53 ± 0.20	93.73 ± 0.21	53.00 ± 4.36	98.07 ± 0.43	H/77.97 ± 3.21
w/o Prior Cross-Attention		94.48 ± 0.15	94.37 ± 0.16	93.19 ± 0.13	93.28 ± 0.14	60.33 ± 3.51	99.67 ± 0.18	C/76.05 ± 0.43
w/o Prior Alignment Loss		92.22 ± 0.58	92.95 ± 0.53	91.57 ± 0.51	91.82 ± 0.50	43.33 ± 3.79	97.29 ± 0.09	C/71.26 ± 0.40
w/o Prior Tokens		92.94 ± 0.21	93.66 ± 0.20	92.48 ± 0.19	92.83 ± 0.20	41.33 ± 3.51	98.91 ± 0.32	H/79.96 ± 3.59
w/o Condition Token		94.65 ± 0.68	95.13 ± 0.70	93.31 ± 0.79	93.65 ± 0.61	42.67 ± 3.79	99.56 ± 0.26	C/72.85 ± 5.22
w/o Auxiliary Pretraining		96.30 ± 0.27	96.33 ± 0.28	95.68 ± 0.10	95.77 ± 0.21	61.33 ± 15.37	99.69 ± 0.11	B/82.10 ± 4.37
**(b) Computational Cost of the BAR Configurations**								
**Configuration**	**Trainable** **Parameters**	**Estimated GFLOPs/Window**		**Peak allocated GPU Memory (MiB)**	**Network Latency,** **Mean ± SD (ms)**		**Pipeline Latency,** **Mean ± SD (ms)**	**Pipeline P95 (ms)**
Eight-block BAR	53,817,385	14.188		225.26	10.89 ± 0.49		22.04 ± 0.29	22.20
Four-block BAR	53,817,385	14.172		221.85	10.95 ± 0.29		22.38 ± 0.39	22.55
No BAR	53,792,041	14.143		216.94	6.23 ± 0.32		17.58 ± 0.29	17.74
**(c) Matched Numerical-Prior Controls on the WC1–WC2 Task**								
**Prior Setting**		**Accuracy (%)**		**Macro-Precision (%)**	**Macro-Recall (%)**			**Macro-F1 (%)**
Nominal priors		99.13 ± 0.04		99.21 ± 0.03	99.05 ± 0.02			99.13 ± 0.02
Uniform +20% shift		95.89 ± 0.21		95.72 ± 0.27	95.10 ± 0.37			95.13 ± 0.34
Fixed permutation		96.53 ± 0.38		96.50 ± 0.32	96.13 ± 0.51			96.16 ± 0.41
Fixed random priors		94.64 ± 0.99		95.11 ± 0.98	93.38 ± 1.38			93.34 ± 1.58

(b) Measurements used FP32 and batch size one. Network latency was measured over 500 repetitions per configuration and includes the complete forward pass with the auxiliary heads retained. Pipeline latency includes CPU preprocessing, data transfer, model execution, and prediction return, excluding acquisition and disk reading, and was measured over 150 repetitions. SD describes timing variability; P95 denotes the 95th-percentile latency. Estimated GFLOPs use two FLOPs per MAC for the principal operations covered by the profiler. (c) All configurations retained the complete architecture and common training protocol. Each controlled prior configuration was applied consistently to both the prior-token inputs and the alignment targets.

**Table 9:** Leave-One-Condition-Out Results on the L Dataset.

Metric	Conditions						Average (%)
	C1 (%)	C2 (%)	C3 (%)	C4 (%)	C5 (%)	C6 (%)	
Accuracy	97.73 ± 0.69	95.20 ± 0.83	97.20 ± 1.73	96.80 ± 1.07	92.00 ± 0.35	93.07 ± 0.96	95.33 ± 0.58
Macro-Precision	97.77 ± 0.65	95.28 ± 0.86	97.27 ± 1.65	96.91 ± 1.05	92.59 ± 0.31	93.37 ± 0.67	95.53 ± 0.56
Macro-Recall	97.73 ± 0.69	95.20 ± 0.83	97.20 ± 1.73	96.80 ± 1.07	92.00 ± 0.35	93.07 ± 0.96	95.33 ± 0.58
Macro-F1	97.74 ± 0.68	95.21 ± 0.85	97.20 ± 1.73	96.80 ± 1.07	91.99 ± 0.38	93.08 ± 0.92	95.33 ± 0.59
*R*_1_	98.00 ± 0.00	93.33 ± 3.33	97.33 ± 1.33	92.00 ± 1.76	100.00 ± 0.00	93.33 ± 1.15	95.67 ± 0.91
*R*_2_	99.33 ± 0.67	100.00 ± 0.00	100.00 ± 0.00	98.67 ± 1.33	98.67 ± 1.33	98.67 ± 0.67	99.22 ± 0.19
*R*_3_	95.33 ± 1.15	92.00 ± 2.40	98.00 ± 1.76	100.00 ± 0.00	84.00 ± 0.67	83.33 ± 3.53	92.11 ± 0.84
*R*_4_	98.67 ± 0.67	96.00 ± 2.67	95.33 ± 2.40	96.00 ± 3.71	87.33 ± 1.76	94.67 ± 3.71	94.67 ± 1.75
*R*_5_	97.33 ± 1.15	94.67 ± 2.91	95.33 ± 4.67	97.33 ± 3.06	90.00 ± 0.67	95.33 ± 2.40	95.00 ± 1.33

**Table 10:** Accuracy Comparison with Baseline Models on the L Dataset.

Model	Conditions						Average (%)
	C1 (%)	C2 (%)	C3 (%)	C4 (%)	C5 (%)	C6 (%)	
PGMV-BAR	97.73 ± 0.69	95.20 ± 0.83	97.20 ± 1.73	96.80 ± 1.07	92.00 ± 0.35	93.07 ± 0.96	95.33 ± 0.58
1D-CNN	71.73 ± 0.93	69.07 ± 4.74	91.73 ± 0.74	87.47 ± 4.64	67.60 ± 1.99	72.27 ± 2.50	76.64 ± 1.90
MpResCNN-BiLSTM-Attention	87.07 ± 3.07	90.80 ± 1.33	88.13 ± 1.79	81.87 ± 3.01	65.47 ± 4.33	75.87 ± 0.58	81.53 ± 0.62
TF-ResNet	93.60 ± 2.81	94.27 ± 4.67	89.87 ± 1.07	92.00 ± 1.97	77.73 ± 1.22	68.40 ± 0.48	85.98 ± 0.43
TF-Swin-Transformer	93.47 ± 4.29	94.13 ± 1.83	90.27 ± 1.79	92.13 ± 0.80	74.93 ± 0.27	78.27 ± 1.31	87.20 ± 0.54
Spec+TF Dual-Branch Fusion	89.20 ± 3.89	92.40 ± 4.24	93.47 ± 0.58	92.53 ± 2.47	65.33 ± 1.62	57.20 ± 1.01	81.69 ± 0.97
PFN–PGSA	34.09 ± 2.95	34.00 ± 1.94	41.24 ± 16.36	42.93 ± 4.28	27.56 ± 3.04	26.00 ± 3.14	34.30 ± 2.81

## Data Availability

The self-collected L dataset generated and analyzed in this study is publicly available in IEEE DataPort under the title “Bearing Fault L Dataset”: https://doi.org/10.21227/9r03-5q43 (accessed on 8 October 2026). The BJTU-RAO dataset used in this study is publicly available; detailed dataset information and access instructions are provided in Reference [1].

## References

[1] Qin Y., Wang Y., Li Z.S., Wang B., Ding A., Wang C., Qin Y., Wang Y. (2025). An in-depth tutorial on BJTU-RAO bogie datasets for fault diagnosis. IEEE Access.

[2] Liu Q., Lai H., Wen B., Hou D., Liao J., Deng C., Dai Z., Chen M., Huang H., Fu J. (2025). A novel method of bearing fault diagnosis for train bogie transmission system based on MpResCNN-BiLSTM model with attention mechanism. IEEE Trans. Intell. Transp. Syst..

[3] Zhu J., Li O., Zhang Y., Chen M., Xing L., Bian S., Liang S. (2025). Time-frequency information multilevel integration and multiscale parallel-fusion network for bearing fault diagnosis. IEEE Sens. J..

[4] Wang B., Lai Y., Li Z., Jiang J., Tan B. (2026). A bearing fault diagnosis method based on weighted differential time–frequency features and a dual-branch interactive fusion network. Sensors.

[5] Yan Y., Shang T., Jia K., Yang S., Cheng H., Li Y., Quan W. (2026). MGFR-ViT: A multi-scale gated feature refinement vision transformer for vibration-based fault diagnosis. Sensors.

[6] Li X., Su H., Xiang L., Yao Q., Hu A. (2024). Transformer-based meta learning method for bearing fault identification under multiple small sample conditions. Mech. Syst. Signal Process..

[7] Song Y., Li Y., Jia L., Zhang Y. (2024). Domain generalization combining covariance loss with graph convolutional networks for intelligent fault diagnosis of rolling bearings. IEEE Trans. Ind. Inform..

[8] Zhao D., Yang F. (2026). Working-condition-indexed generative domain generalization for intelligent fault diagnosis under unseen conditions. Sensors.

[9] Zhou J., Luo J., Qi J., Qin Y. (2026). Knowledge library network for non-exemplar incremental remaining useful life prediction. IEEE/ASME Trans. Mechatron..

[10] Wang L., Qi J., Qin Y. (2026). A contrastive cluster zero-shot model for cross-type fault diagnosis of bearings. Adv. Eng. Inform..

[11] Qi J., Chen Z., Chen P., Kong Y., Qin Y., Li W. (2026). TimeGPT for mechanical system degradation assessment: A large time series model approach. Chin. J. Mech. Eng..

[12] Zhou Y., Liang M., Yue X. (2024). Deep residual learning for acoustic emission source localization in a steel-concrete composite slab. Constr. Build. Mater..

[13] Selami S., Ataya S., Karmi Y., Debbah Y., Guilbault R., Khan R., Boumediri H. (2026). Hybrid IGWO-DNN approach for bearing fault diagnosis using time domain features from empirical mode decomposition. Int. J. Adv. Manuf. Technol..

[14] Cheng W., Liu X., Xing J., Chen X., Ding B., Zhang R., Zhou K., Huang Q. (2023). AFARN: Domain adaptation for intelligent cross-domain bearing fault diagnosis in nuclear circulating water pump. IEEE Trans. Ind. Inform..

[15] Jian C., Yin Z., Zhang Y., Ao Y., Chen H., Wang H. (2026). A physics-informed generalization framework for cross-condition bearing fault diagnosis with limited labeled data. Inf. Sci..

[16] Shi Z., Wang K. (2026). Physics-guided frequency normalization enables cross-speed inner-race bearing fault transfer diagnosis. Electronics.

[17] Sim T.-S., Aronwora N.C., Hur J.-W. (2026). A physics-guided dual-sensor framework for bearing fault diagnosis in PMDC motor drives. Sensors.

[18] Kimi Team (2026). Attention residuals. arXiv.

[19] Randall R.B., Antoni J. (2011). Rolling element bearing diagnostics—A tutorial. Mech. Syst. Signal Process..

[20] Allen J.B., Rabiner L.R. (1977). A unified approach to short-time Fourier analysis and synthesis. Proc. IEEE.

[21] Vaswani A., Shazeer N., Parmar N., Uszkoreit J., Jones L., Gomez A.N., Kaiser Ł., Polosukhin I. Attention is all you need. Proceedings of the 31st Conference on Neural Information Processing Systems (NIPS 2017).

[22] Chen T., Kornblith S., Norouzi M., Hinton G. A simple framework for contrastive learning of visual representations. Proceedings of the 37th International Conference on Machine Learning (ICML 2020).

[23] Lin T.-Y., Goyal P., Girshick R., He K., Dollár P. Focal loss for dense object detection. Proceedings of the 2017 IEEE International Conference on Computer Vision (ICCV).

[24] Loshchilov I., Hutter F. Decoupled weight decay regularization. Proceedings of the 7th International Conference on Learning Representations (ICLR 2019).

[25] Zhang X., Xie J., Wang F. (2026). A feature extraction interpretability research of one-dimensional convolutional neural networks in mechanical fault diagnosis. Proc. Inst. Mech. Eng. Part C J. Mech. Eng. Sci..

[26] Wang C., Li Y., Wang T., Yang C., Liang C. (2026). Bearing fault diagnosis method based on SE-ResNet-50 and Swin Transformer. Modul. Mach. Tool Autom. Manuf. Tech..

[27] Zeng X., Long J., Wei Y., Wu Y. (2025). Fault diagnosis method for rolling bearings based on VMD-CWT and Swin Transformer. Mach. Build. Autom..

[28] Liu J., Li G., Tang X., Zhou P., Zhang L. (2026). Bearing fault diagnosis method based on time-frequency dual-flow graph attention network. J. Railw. Sci. Eng..

[29] Xie J., Xu Y., Meng Z., Liang M., Zhou W., Zhou Y., Liu C., Schlangen E., Šavija B. (2026). Bayesian-driven modulation of recoverable strain in compliant auxetic cementitious composites for optimal energy harvesters. Mater. Des..

